# Overexpression of CD157 Contributes to Epithelial Ovarian Cancer Progression by Promoting Mesenchymal Differentiation

**DOI:** 10.1371/journal.pone.0043649

**Published:** 2012-08-20

**Authors:** Simona Morone, Nicola Lo-Buono, Rossella Parrotta, Alice Giacomino, Giulia Nacci, Alfredo Brusco, Alexey Larionov, Paola Ostano, Maurizia Mello-Grand, Giovanna Chiorino, Erika Ortolan, Ada Funaro

**Affiliations:** 1 Department of Genetics, Biology and Biochemistry, University of Torino, Torino, Italy; 2 Laboratory of Cancer Genomics, Fondazione Edo ed Elvo Tempia Valenta, Biella, Italy; 3 Research Center on Experimental Medicine (CeRMS), University of Torino, Torino, Italy; Cedars-Sinai Medical Center, United States of America

## Abstract

Epithelial ovarian carcinoma (EOC) is an aggressive tumor often diagnosed at an advanced stage, when there is little or no prospect of cure. Despite advances in surgical and chemotherapeutic strategies, only marginal improvements in patient outcome have been obtained. Hence, unraveling the biological mechanisms underpinning EOC progression is critical for improving patients’ survival. Recently, we reported that CD157 (an ectoenzyme regulating leukocyte diapedesis) is expressed in EOC and that high expression of the molecule is negatively correlated with the disease outcome in patients. Here, we demonstrate that forced overexpression of CD157 in OVCAR-3, TOV-21G, A2780 and OV-90 ovarian cancer cell lines promotes morphological and phenotypic changes characterized by disruption of intercellular junctions, downregulation of epithelial markers and upregulation of mesenchymal ones. These changes in cell shape and phenotype bring to reduced sensitivity to anoikis, increased anchorage-independent growth, cell motility and mesothelial invasion. Conversely, knockdown of CD157 in OV-90 and OC314 cells reverts the mesenchymal phenotype and reduces the cells’ migratory potential. Transcriptome profiling analysis highlighted 378 significantly differentially expressed genes, representing the signature of CD157-overexpressing OVCAR-3 and OV-90 cells. The modulation of selected genes translates into alteration of protein expression that give cells a highly malignant phenotype. The overall picture deduced from the analysis of the modulated transcripts is that high expression of CD157 strengthens a number of biological processes favoring tumor progression (including development and cell motility), and weakens several biological processes hindering tumor progression (such as apoptosis, cell death and response to stress). Together, these findings implicate CD157 in the progression of EOC to metastatic disease and suggest that CD157 may represent a valuable therapeutic target.

## Introduction

Epithelial ovarian cancer (EOC) is an aggressive and lethal gynecological malignancy. Over 70% of patients present with advanced disease and, despite aggressive treatment, the 5-years survival rate of patients with EOC is below 50%. This poor prognosis results from the difficulty of diagnosis in the early clinical stages and the lack of an effective therapy for advanced-stage tumors. Understanding the biological mechanisms regulating the progression of EOC is therefore critical for devising new treatment options and improving patients’ survival.

EOC is thought to arise from the ovarian surface epithelium that lines the ovary. EOC cells can shed from the primary tumor and, because no anatomical barrier is present, spread directly throughout the peritoneal cavity and then disseminate mainly via the lymphatic system, developing the necessary defense mechanisms for survival under anchorage-independent conditions [1]. In the tumor environment, localized proteolytic degradation of the extracellular matrix (ECM) facilitates the migration of floating cells, allowing them to anchor to the mesothelium and subsequently invade it, establishing tumors at secondary sites. Tumor dissemination implies a phenotypic conversion of epithelial cells, which are not motile, into mesenchymal cells. This process has remarkable similarities with the epithelial-mesenchymal transition (EMT) occurring during embryonic development [2]. Indeed, type 3 or oncogenic EMT is increasingly recognized as a dynamic and transient mechanism whereby cells in primary non-invasive tumors acquire properties essential for migration, invasion, metastatic dissemination and resistance to apoptosis [3]. The EMT program can be induced by a variety of contextual signals that cells might experience in the tumor microenvironment; regardless of the trigger signals, activation of the EMT is associated with poor clinical outcome in different types of tumors, including ovarian cancer [4]. Cell surface molecules involved in the control of processes such as cell-cell, cell-ECM adhesion, localized intraperitoneal migration and invasion of the peritoneum by floating cells or cell aggregates (spheroids) are believed to play a leading role in EOC progression and, ultimately, in patients’ outcome.

CD157/BST-1, a GPI-anchored member of a family of NADase/ADP-ribosyl cyclase, is an ectoenzyme that cleaves extracellular nicotinamide adenine dinucleotide (NAD^+^), generating cyclic ADP ribose (cADPR) and ADPR [5], [6]. In addition, CD157 establishes functional and structural interactions with other transmembrane molecules thus acquiring the ability to transduce intracellular signals [7]–[9]. Although CD157 was initially characterized as a stromal [10] and myeloid surface glycoprotein [11] involved in the control of cell migration and diapedesis [12], we recently demonstrated that CD157 is also expressed by >90% of primary EOC and that high levels of CD157 are associated with rapid tumor relapse in patients with EOC. Consistently with these findings, inhibition of CD157 activity, by a specific monoclonal antibody (mAb) *in vitro* or by its weak expression in patients, is associated with reduced tumor cell invasion and migration. The association of CD157 with EOC aggressiveness has been further substantiated by the observation that exogenous expression of CD157 in scarcely motile, CD157-negative EOC cells substantially increases cell motility, a prerequisite for tumor cells invasion into surrounding tissues [13].

The implication of CD157 in tumor cell motility and invasiveness, and its association with poor outcome in ovarian cancer patients, prompted us to further investigate its biological role in EOC progression using engineered ovarian cancer cell lines as an experimental model. The ultimate goal was to understand how the function of CD157 might contribute to a more aggressive ovarian cancer and whether CD157 might be helpful in assisting the management of these patients.

## Materials and Methods

### Cell Lines and Reagents

The human EOC cell lines OVCAR-3 and OV-90 and the non-malignant pleural mesothelial cell line Met-5A were purchased from American Type Culture Collection (ATCC, Manassas, VA). The EOC cell lines TOV-21G and A2780 were provided by M.F. Di Renzo (University of Turin, Italy) and OC314 was provided by S. Ferrini (Institute for Cancer Research and Treatment, Genoa, Italy). The anti-CD157 mAb (SY/11B5, kindly provided by F. Malavasi, University of Turin, Italy) was produced in the authors’ laboratories and affinity purified on protein G (Sigma-Aldrich). The Alexa-488 labeled F(ab′)_2_ fraction of goat antibodies to mouse IgG or to rabbit IgG were from Molecular Probes (Milan, Italy). Anti-E-cadherin mAb was from BD Biosciences (Milan, Italy), anti-Snail, anti-Zeb1, anti-EpCAM, anti-VCAN, anti-BMP7, anti-tubulin, anti-β-catenin, anti-N-cadherin and anti-β-actin-horseradish peroxidase (HRP) mAb were from Santa Cruz Biotechnologies (Santa Cruz, CA), anti-lamin B1 was from Abcam (Cambridge, UK). TRITC-labelled phalloidin used to detect F-actin was from Sigma-Aldrich. GM6001 (matrix metalloproteases inhibitor) was from Enzo Life Science (Vinci Biochem, Vinci, Italy).

### CD157 Gene Transfection and ShRNA Lentiviral Particle Transduction

Cells were transfected with the eukaryotic expression vector pcDNA3.1 containing the cDNA for full-length CD157 or no insert (mock), as described [13]. Cells were grown in RPMI-1640 or MCDB131/M199 (vol/vol) culture medium (Sigma-Aldrich, Milan, Italy) supplemented with 10% fetal calf serum (FCS, Biochrom Seromed, Milan, Italy). Cells were maintained at 37°C and 5% CO2 and tested for *Mycoplasma* contamination.

CD157 expression in OV-90 and OC314 cells was silenced by lentiviral delivery of pLV-puro (Biosettia, San Diego, CA) encoding a short-hairpin RNA (shRNA) targeting BST-1 mRNA (target sequences 5′-GAGTCAGACTGCTTGTATA-3′ (shCD157) and 5′-CCTGAGCGATGTTCTGTAT-3′ (shCD157#2); scrambled sequence 5′-TTCTCCGAACGTGTCACGTT-3′). Particles were generated as previously described [14]. Cells were incubated with appropriate lentiviral supernatants and Polybrene (8 µg/ml, Sigma-Aldrich). Transduced cells underwent selection in 2 µg/ml puromycine (Santa Cruz Biotechnologies) for 3 days.

**Figure 1:**
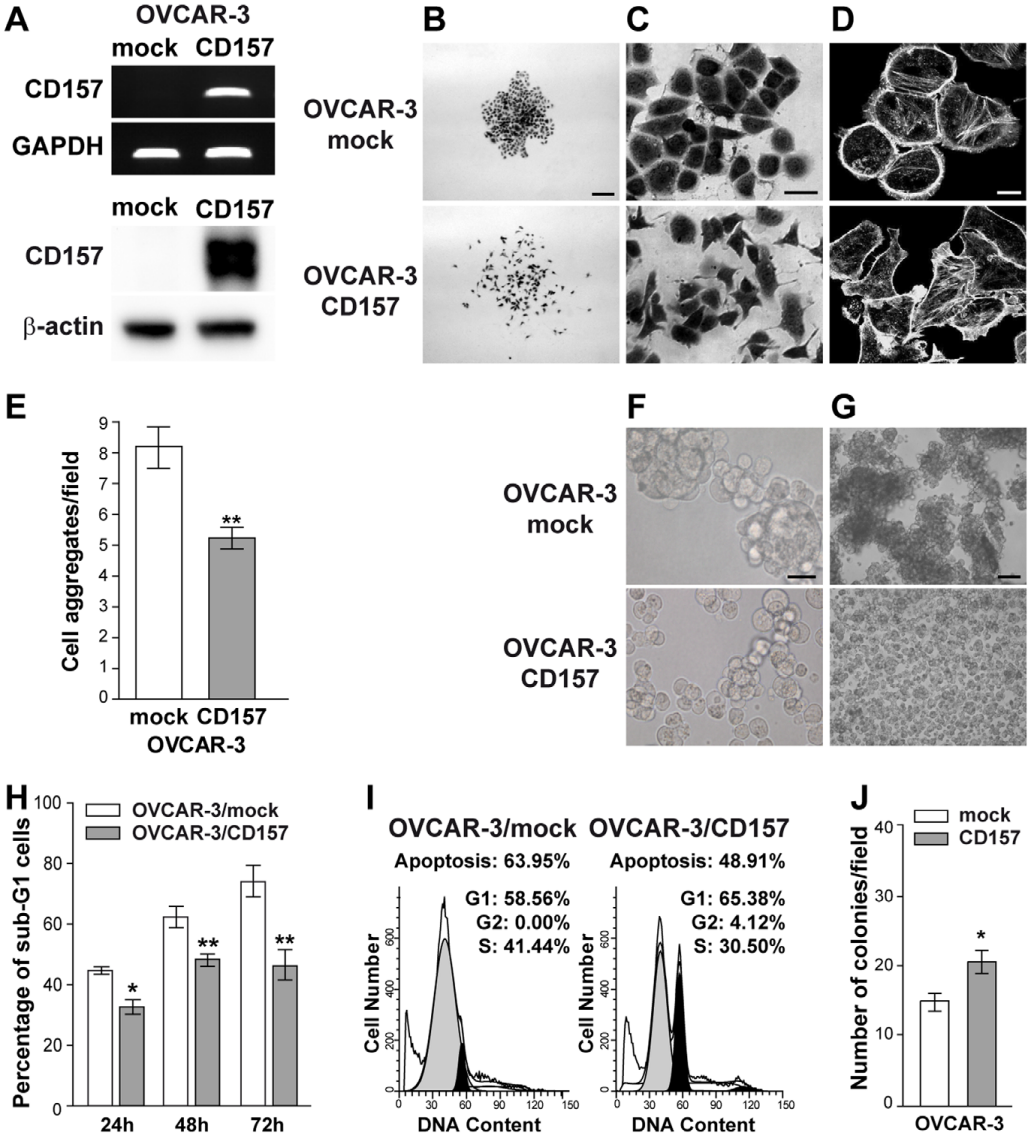
Ectopic expression of CD157 alters cell morphology, cell-cell interactions and anoikis in OVCAR-3 cells. (A) sqRT-PCR of ectopic CD157 expression in OVCAR-3 cells (top). GAPDH was used as an internal control. Western blot analysis of CD157 in OVCAR-3/CD157 and OVCAR-3/mock cells (bottom). The anti-β-actin mAb was used as a loading control. (B–C) Morphology of OVCAR-3/mock and OVCAR-3/CD157 cells. Representative colonies visualized after crystal violet staining using an IX70 inverted microscope equipped with a UC30 camera and the CellF analysis software (Olympus Biosystems) are shown. (B, scale bar: 200 µM; C, scale bar: 20 µM). (D) Confocal microscopy analysis of F-actin. Cells were grown on gelatin-coated coverslips, fixed with 2% PFA, permeabilized with 0.2% Triton-X 100 and stained with phalloidin-TRITC. Samples were analyzed using an Olympus FV300 laser scanning confocal microscope. Cells were imaged using a 60× oil immersion objective (1.4 NA). (Scale bar: 20 µM). Microphotographs in B, C, and D were then reproduced in black and white. (E) OVCAR-3 cells were subjected to a cell-cell adhesion assay and clusters (>5 cells) were counted in 20 different fields/dish. Results represent the mean ± SEM of four independent experiments. **P<0.01, two-tailed t test. (F) Aggregates generated using the hanging drop method and overnight incubation at 37°C were mechanically dispersed and then photographed under phase contrast microscope (scale bar: 50 µM). (G) Induction of anoikis in OVCAR-3/CD157 and OVCAR-3/mock cells after 72 h of culture on poly-HEMA-coated plates. Phase-contrast microscopy images show the formation of large floating aggregates in OVCAR3/mock cells and small aggregates or single isolated cells in OVCAR3/CD157 cells (scale bar: 200 µM). (H) After 24, 48 and 72 h of anchorage-independent growth, cells were fixed, permeabilized, stained with propidium iodide and analyzed with a FACSCanto. Data analysis was performed with ModFit LT™ cell cycle analysis software. Anoikis in OVCAR-3/mock and OVCAR-3/CD157 cells was determined by measuring the percent of sub-G1 cells. Results represent the mean ± SEM of three independent experiments. *P<0.05; **P<0.01, two-tailed t test. (I) Representative histograms of cell cycle status of mock and CD157-positive OVCAR-3 cells after 48 h of anchorage-independent growth. (J) Anchorage-independent growth of OVCAR-3/CD157 and mock cells was analyzed by soft agar colony formation assay. Graph represents average number of colonies formed from three independent experiments ± SEM after 3 weeks incubation of cells in soft agar. *P<0.05, two-tailed t test.

### Western Blot Analysis

Total cell lysates were obtained by incubation in RIPA lysis buffer (50 mM Tris HCl, 150 mM NaCl, 1% NP-40, 0.5% Sodium Deoxycholate, 1 mM EDTA, 0.1% SDS supplemented with 1 mM Na_3_VO_4_, 5 mM NaF, 50 µg/ml aprotinin and leupeptin). Cytosolic extracts were obtained by incubating cells for 10 minutes at 4°C with hypotonic buffer solution containing 20 mM Tris-HCl pH 7.4, 10 mM NaCl, 3 mM MgCl_2_ and 50 µg/ml Protease Inhibitor Cocktail (Sigma-Aldrich). The suspension was treated with a 0.5% NP40 solution and the obtained homogenate was centrifuged (3.000 rpm, 10 minutes at 4°C). Nuclear extracts were prepared by incubating the pellet obtained as described above in RIPA Lysis buffer for 30 minutes. After 30 minutes centrifugation at 14.000 rpm at 4°C, protein concentration was determined using the Bradford assay (Bio-Rad Laboratories). Western blotting was performed as previously described [13]. Briefly, equal amounts (30 µg) of protein extracts from cells were separated by 10% SDS-polyacrylamide gel electrophoresis (PAGE) under non-reducing conditions and electro-transferred on a polyvinylidene difluoride (PVDF) membranes, then blocked and probed with the indicated mAb. After incubation with the appropriate HRP-conjugated antibodies (Santa Cruz Biotechnologies), the immunoreactive bands were detected by enhanced chemiluminescence (Perkin Elmer, Monza, Italy). Images were captured with a ChemiDoc™ XRS+ System and densitometry analysis was performed with Image Lab™ Software (Bio Rad, Milan, Italy).

### Cell Colony Scattering Assay and Soft Agar Colony Formation

Cells (500/well) were seeded in 6-well plates and maintained in culture medium supplemented with 5% FCS for 14 days, then washed, fixed in methanol for 30 minutes, stained with crystal violet (Sigma-Aldrich) and visualized using an IX70 inverted microscope equipped with a UC30 camera and the CellF analysis software (Olympus Biosystems).

In soft agar assays 6×10^2^ cells were mixed with 0.45% agar solution in RPMI containing 10% FCS and layered on top of 0.9% base agar layer in 24-well plates. Assays were performed in triplicate. After 2–3 weeks at 37°C in a 5% CO_2_ incubator, colonies were visualized with an inverted microscope and counted.

### Cell-cell Adhesion and Cell Aggregation Assays

Cell-cell adhesion experiments were performed as described [15]. Briefly, single cell suspensions were seeded on 0.5% agarose-coated culture dishes (1 ml/well, 30 minutes at 37°C) to prevent cell adhesion, and slowly shaken for 2 h at 37°C.

Cell aggregation assays were performed using the hanging drop method, as described [16]. Briefly, cells (5×10^3^/25 µl of RPMI 1640 medium with 5% FCS) were seeded onto the inner surface of the lid of a Petri dish. To prevent evaporation, 10 ml phosphate buffered saline (PBS) were placed into the dish. After overnight incubation at 37°C, cell aggregates were mechanically dispersed and photographed using a phase-contrast microscope.

### Anoikis Assay

Cells (5×10^5^/well) were cultured on 20 mg/ml poly-HEMA (polyhydroxyethylmethacrylate, Sigma-Aldrich)-treated 6-well plates for 24 to 72 h. Then, cell aggregates were dispersed and the cells were fixed with ice-cold 75% ethanol (vol/vol) overnight at 4°C. Cells were treated with 100 µg/ml RNase A (Sigma-Aldrich, 30 minutes at 37°C), stained with 10 µg/ml propidium iodide (10 minutes at 4°C) and samples were analyzed with a FACSCanto (Becton Dickinson, Mountain View, CA, USA). Data analysis was performed using ModFit LT™ cell cycle analysis software (Verity Software House, Topsham, ME). Anoikis was determined by measuring the percent of sub-G1 cells.

### Immunofluorescence Staining and Confocal Microscopy

In confocal microscopy experiments, cells were grown to subconfluence on gelatin-coated coverslips, fixed with 4% paraformaldehyde (PFA) for 30 minutes at 20°C, washed and permeabilized with 0.1% Triton X-100 in phosphate-buffered saline with 1% normal goat serum and 1% BSA for 15 minutes at 20°C. Cells were incubated with the indicated primary antibodies followed by Alexa Fluor-488-conjugated secondary antibodies. The samples were analyzed with an Olympus FV300 laser scanning confocal microscope equipped with a Blue Argon (488 nm) laser, a Green Helium Neon (543 nm) laser, and FluoView 300 software (Olympus Biosystems, Hamburg, Germany). Cells were imaged using a 60× oil immersion objective (1.4 NA) and 10× ocular lens. Nomarski images were obtained by differential interference contrast (DIC) optical components installed on an IX71 inverted microscope. Semiquantitative analysis of E-cadherin junctional staining was performed by counting a minimum of 10 fields per sample (at least 200 cells overall) and scoring as positive the number of cells with two remaining fluorescent cell-cell borders.

### RNA Extraction and Reverse Transcriptase-PCR

Total RNA (2 µg) extracted from 70–80% confluent cultures using TRIZOL® reagent (Invitrogen, S. Giuliano Milanese, Italy) was reverse-transcribed with the M-MLV Reverse Transcriptase (Invitrogen) and Oligo-dT primers. cDNA was amplified using KAPA2G Fast HotStart DNA Polymerase (Kapa Biosystems, Cambridge, MA). Each cycle consisted of denaturation at 94°C for 10 seconds, annealing for 10 seconds and extension at 72°C for 1 second. In semi-quantitative analysis (sqRT-PCR), the appropriate number of cycles for remaining within the exponential phase was determined for each substrate. The primers used are reported in Table S1. PCR products were then analyzed by agarose gel electrophoresis.

### SYBR Green Real-time RT-PCR (qRT-PCR)

Total RNA was extracted using the RNeasy mini kit (Qiagen, Milan, Italy) according to the manufacturer’s directions. qRT-PCR was performed using SYBR Green JumpStart Taq ReadyMix (Sigma-Aldrich) and an ABI 7500 Fast Sequence Detection System (Applied-Biosystems, Foster City, CA, USA). PCR cycling conditions were performed for all samples as follows: 95°C for 10 minutes, followed by 40 cycles at 95°C for 15 seconds and 60°C for 1 minute. The primers used are listed in Table S2. PCR reactions for each template were done in triplicate in 96-well plates. The comparative CT method (Applied Biosystems) was used to determine gene expression in CD157-transfected relative to the value observed in the corresponding control cells, using TBP as normalization control.

**Figure 2:**
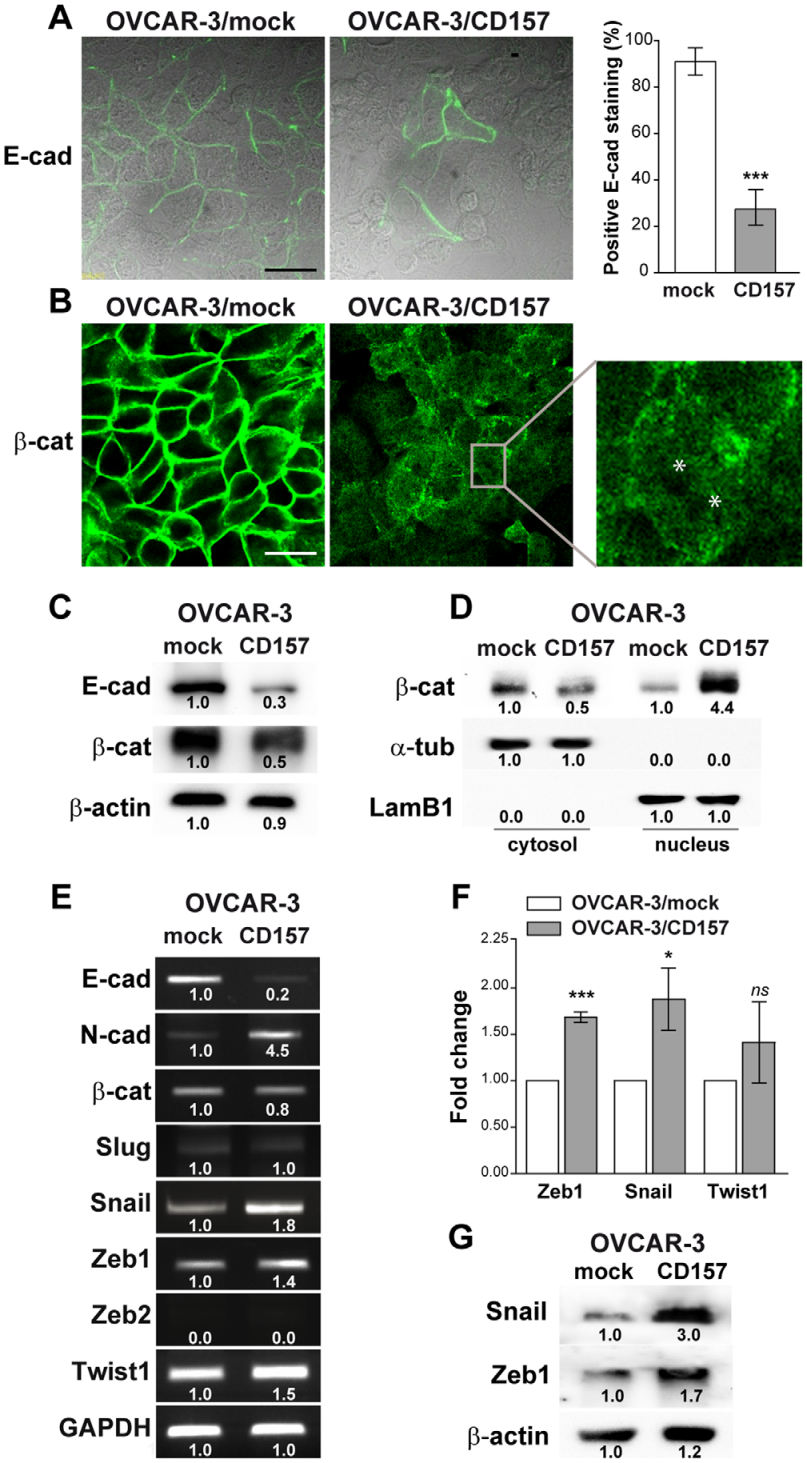
CD157 overexpression alters the expression of epithelial and mesenchymal markers. (A) Confocal microscopy analysis of E-cadherin and (B) β-catenin expression in OVCAR-3/CD157 and mock cells. Cells were grown on a gelatin-coated coverslip, fixed, permeabilized and stained with anti-E-cadherin, and anti-β-catenin antibodies followed by secondary Alexa Fluor-488-labelled antibody. Samples were analyzed with an Olympus FV300 laser scanning confocal microscope and by Nomarski differential interference contrast (DIC) optics. For E-cadherin, a fluorescence image merged with DIC image is shown (scale bar: 50 µM). Semiquantitative analysis of E-cadherin junctional staining was determined by counting a minimum of 10 fields/sample (at least 200 cells overall) and scoring as positive cells with two remaining fluorescent intercellular borders. For β-catenin, a fluorescent image is shown. Inset: amplified view of an individual OVCAR-3/CD157 cell exhibiting diffuse β-catenin staining in the plan of focus cutting through the nucleus. Asterisks correspond to nucleoli. (C) Western blotting for E-cadherin and β-catenin in OVCAR-3/CD157 and mock cells. Densitometry quantifies the expression level of E-cadherin and β-catenin relative to β-actin. (D) β-catenin levels in nuclear and cytoplasmic fractions of OVCAR-3/mock and OVCAR-3/CD157 cells were determined by western blot analysis. α-tubulin and lamin B1 (LamB1) were used as cytoplasmic and nuclear loading controls, respectively. Densitometry quantifies the expression level of β-catenin relative to the proper control. (E) sqRT-PCR for E-cadherin, N-cadherin, β-catenin and for E-cadherin transcriptional repressors in OVCAR-3/CD157 and mock cells. Densitometry quantifies the levels of expression of E-cadherin repressors relative to GADPH. (F) qRT-PCR for Zeb1, Snail and Twist1. The comparative CT method was used to determine gene expression in CD157-transfected cells relative to the value observed in the mock cells, using TBP as normalization control. Histograms report the means ± SEM of three qRT–PCR independent experiments, each conducted in triplicate. *P<0.05, ***P<0.001; *ns*, not significant; two-tailed t test. (G) Western blot analysis showing the level for Snail and Zeb1 in OVCAR-3/mock and OVCAR-3/CD157 cells. Densitometry quantifies the expression level of both proteins relative to β-actin. Results shown in each panel are representative of three independent experiments with similar results.

### Wound-healing Assay

Cells were seeded in six-well plates to confluence and a scratch was then made across the monolayer, as previously described [13]. Images of the wounded area were recorded at the times indicated. Each experiment was performed in quadruplicate and repeated at least three times.

### Transmesothelial Migration Assays and Confocal Microscopy Analysis

Met-5A cells (2×10^5^) were labeled using a CellBrite™ Red Cytoplasmic Membrane Staining kit according to the manufacturer’s directions (Biotium Inc. Hayward, CA), then seeded on fibronectin-coated (10 µg/ml) coverslips and allowed to grow to confluence. Ovarian cancer cells (1.5×10^5^) were stained with 5 µM CFSE (Carboxyfluorescein succinimidyl ester, Molecular Probes) and seeded on the Met-5A monolayer for 6 h (OVCAR-3 cells) or 2.5 h (OV-90 cells) at 37°C. Non-adherent tumor cells were carefully removed, the sample fixed with 2% PFA and then analyzed using an Olympus FV300 laser scanning confocal microscope. Cells were imaged using a 60× oil immersion objective (1.4 NA) by sequential scanning of the XY planes recorded along the Z-axis (step size: 1.25 µm). Cells were counted on the top, median and bottom stage and the ratio of cells on the bottom stage to total cells represents the percentage of cells that migrated through the monolayer [17]. Where indicated, cells were treated for 1 h with GM6001 (25 µg/ml) before seeding onto the Met-5A mesothelial cell monolayer.

### Gelatin and Casein Zymography Assays

OVCAR-3 and OV-90-transfected cells were seeded in 48-well plates and grown to 80% confluence, then incubated for an additional 48 h in FCS-free medium. Matrix metalloproteinases (MMPs) MMP2 and MMP9 activity in the conditioned medium was analyzed using 10% SDS-PAGE containing 1 mg/ml gelatin (gelatin zymography) [18], and MMP7 activity was visualized using 12% SDS-PAGE containing 1 mg/ml casein (casein zymography) [19]. Gels were stained with Coomassie brilliant Blue G-250 to visualize protease activity. Images were captured with a ChemiDoc™ XRS+ System and densitometry analysis was performed using Image Lab™ Software.

### Spheroid Disaggregation Assay

Spheroids were generated with the hanging drop method and their disaggregation on fibronectin-coated plates (10 µg/ml) was measured after 12 h incubation at 37°C, as described elsewhere [13]. Briefly, 96-well plates were coated with 10 µg/ml fibronectin and blocked with BSA (1 mg/ml) for 1 h at 37°C. Spheroids (8–10 spheroids/well) were seeded in serum-free RPMI-1640 medium. To track individual spheroids over time, each well was photographed 30 minutes after seeding (time 0) and after 12 h. The pixel area of the spheroids was measured at time 0 and 12 h, and the fold change in area was calculated as the ratio between the pixel area of the spheroids at 12 h and at time 0.

### Microarray Hybridization, Data Collection and Analysis

Total RNA was extracted from duplicate 70–80% confluent control cell lines (OVCAR-3/mock and OV-90/mock) and testing cell lines (OVCAR-3/CD157 and OV-90/CD157), microarray probe preparation, hybridization, scanning and image analysis were performed as previously described [20]. Two replicates, with dye swap, were performed for each sample. Raw data elaboration was carried out with Bioconductor [21], using R statistical language [22] applying a cut-off on the P-value, adjusted for multiple testing by the Benjamini-Hochberg approach (<0.01), followed by filtering on expression level (logFC >1 or <−1 in at least one cell line, excluding transcripts with opposite sign). This criterion allowed the identification of transcripts with concordant modulation and took into account intrinsic biological differences of distinct cell lines that might be reflected in the amplitude of fold changes.

Gene ontology, canonical pathway, and functional network analyses were performed using both the DAVID Knowledgebase [23] and MetaCore software from GeneGo Inc., applying a cut-off on enrichment P-values (<0.05). Gene expression data sets have been deposited into the Gene Expression Omnibus (GEO) database, ID: GSE36364.

(http://www.ncbi.nlm.nih.gov/geo/query/acc.cgi?token=flktjkcsuyygcrm&acc=GSE36364).

### Statistical Methods and Data Analysis

Unless otherwise indicated, values are expressed as means ± SEM. Comparisons between two groups were carried out using an unpaired two-sided Student’s *t* test for normal distributed variables. Statistical analyses were performed using SPSS Statistics 17 software (Chicago, IL) and GraphPad Prism 5 software (San Diego, CA). All statistical tests were two-sided. For all analyses, differences were considered significant at P<0.05 (*P<0.05, **P<0.01, ***P<0.001 *versus* control) and *ns* for not significant.

**Figure 3:**
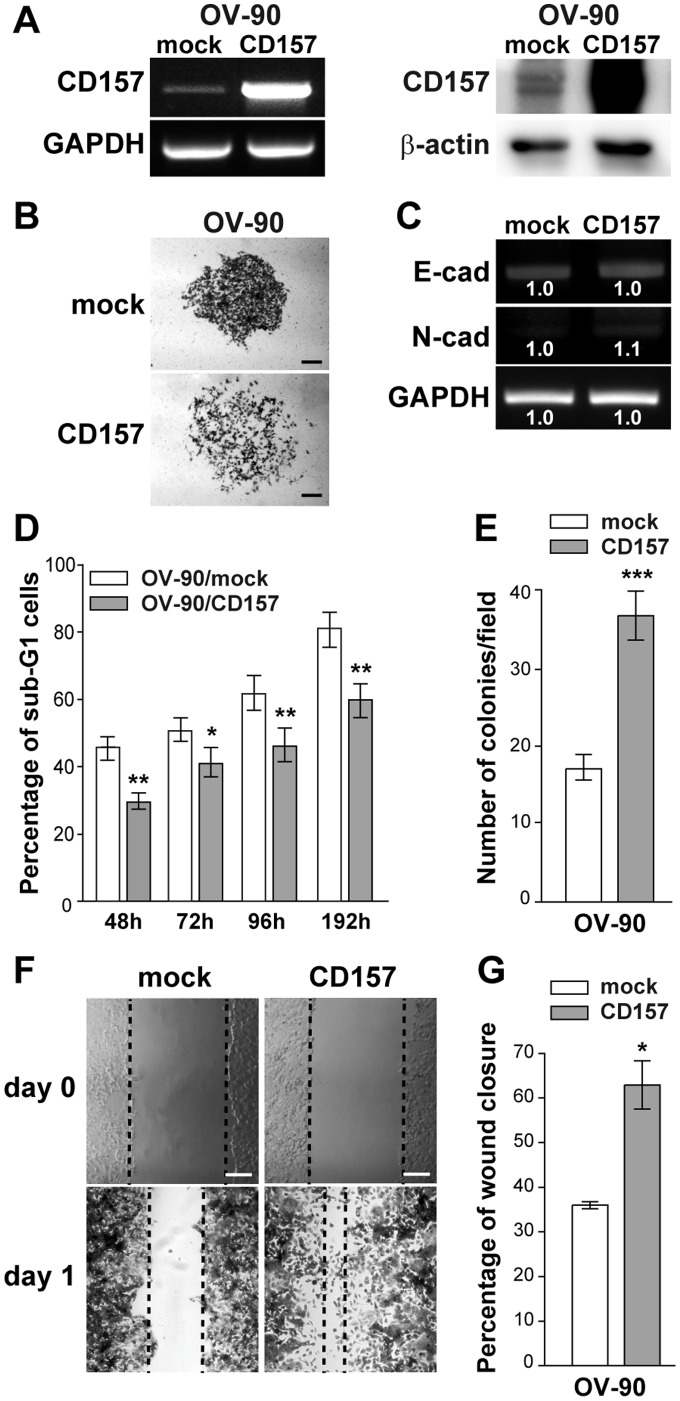
CD157 overexpression protects OV-90 cells from anoikis and enhances motility. (A) sqRT-PCR (left) and western blot analysis (right) of CD157 in OV-90/CD157 and OV-90/mock cells. GAPDH and β-actin were used as internal controls, respectively. (B) Morphology of colonies formed by OV-90/mock and OV-90/CD157 cells. Representative colonies visualized after crystal violet staining are shown. Scale bar: 200 µM. (C) sqRT-PCR analysis of E-cadherin and N-cadherin in OV-90/mock and OV-90/CD157 cells. Densitometry quantifies the levels of mRNA expression of the indicated molecules relative to GAPDH. (D) Effect of CD157 overexpression on anoikis. After 48, 72. 96 and 192 h of anchorage-independent growth, cells were fixed, stained with propidium iodide and analyzed with a FACSCanto. Anoikis in OV-90/mock and OV-90/CD157 cells was determined by measuring the percent of sub-G1 cells. Results represent the mean ± SEM of three independent experiments. *P<0.05; **P<0.01, two-tailed t test. (E) Anchorage-independent growth of OV-90/CD157 and mock cells was analyzed by soft agar colony formation assay. Graph represents average number of colonies formed from three independent experiments ± SEM after 3 weeks incubation of cells in soft agar. ***P<0.001, two-tailed t test. (F) Effect of CD157 expression on cell migration in a scratch-wound assay in OV-90/CD157 and mock cells. Cells were grown as monolayers, wounded, and photographed at time 0 and at 24 hr. Wound edges are indicated by black dashed lines (scale bar: 200 µM). (G) The ability of cells to close the wound was calculated by measuring 20 randomly chosen distances along the wound edge at time 0 and at 24 hr. Results represent the percentage reduction of the average wound width and are expressed as the mean ± SEM of three independent experiments. *P<0.05; two-tailed t test.

**Figure 4:**
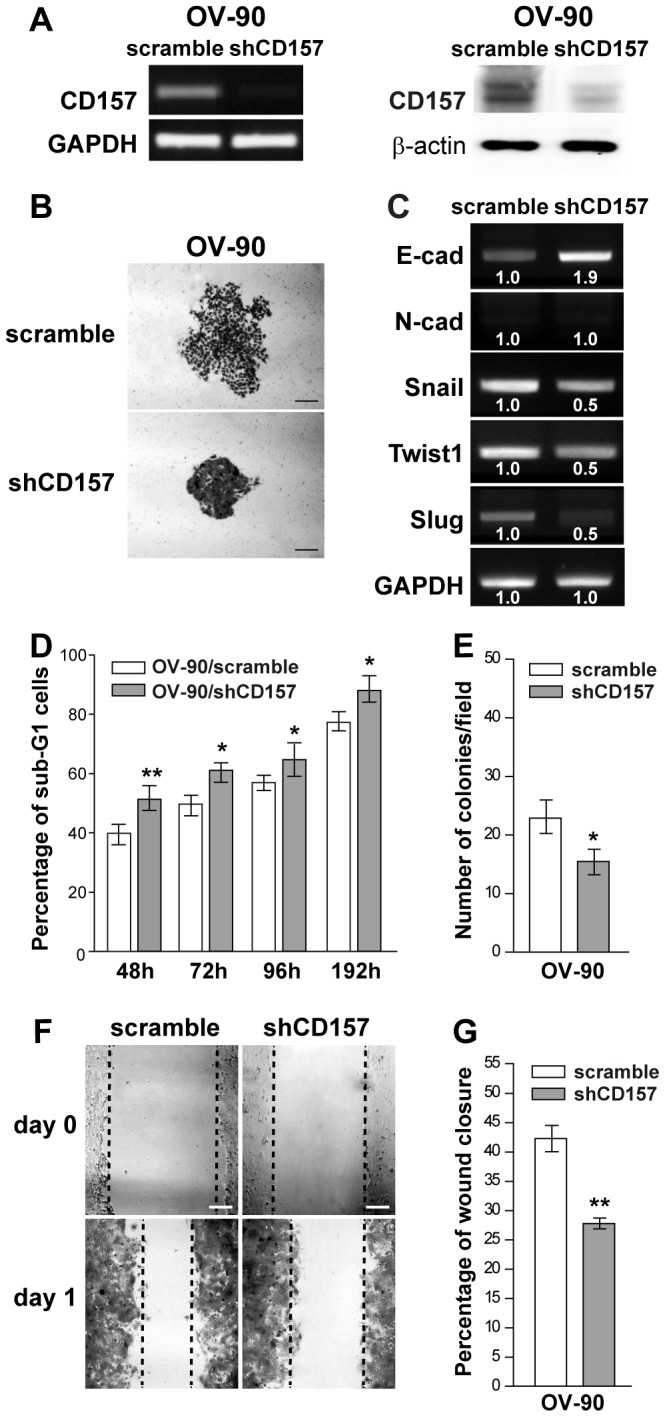
Morphological and functional modifications induced by CD157 knockdown in OV-90 cells. (A) sqRT-PCR (left) and western blot analysis (right) showing OV-90 cells retrovirally transduced with a shRNA that targets the human CD157 mRNA, resulting in efficient knockdown of CD157 expression. GAPDH and β-actin were used as internal controls, respectively. (B) Morphology of colonies formed by OV-90/scramble and OV-90/shCD157 cells. Representative colonies visualized after crystal violet staining are shown. Scale bar: 200 µM. (C) sqRT-PCR for E-cadherin, N-cadherin and Snail, Twist1 and Slug transcriptional repressors in OV-90/scramble and OV-90/shCD157 cells. Densitometry quantifies the levels of mRNA expression of the indicated molecules relative to GAPDH. (D) Anoikis assay. After 48, 72, 96 and 192 h under anchorage-independent growth, cells were fixed, stained with propidium iodide and analyzed with a FACSCanto. Data analysis was performed with ModFit LT™ cell cycle analysis software. Anoikis in OV-90/scramble and OV-90/shCD157 cells was determined by measuring the percent of sub-G1 cells. Results represent the mean ± SEM of three independent experiments. *P<0.05; **P<0.01, two-tailed t test. (E) Anchorage-independent growth of OV-90/scramble and OV-90/shCD157 cells was analyzed by soft agar colony formation assay. Graph represents the average number of colonies/field formed from three independent experiments ± SEM after 3 weeks incubation of cells in soft agar. *P<0.05, two-tailed t test. (F) Effect of CD157 knockdown on OV-90 cell migration in a scratch-wound assay. Cells were grown as monolayers, wounded, and photographed at time 0 and at 24 h (scale bar: 200 µM). Wound edges are indicated by black dashed lines. (G) The ability of OV-90/scramble and OV-90/shCD157 cells to close the wound was calculated by measuring 20 randomly chosen distances along the wound edge at time 0 and at 24 h. Results represent the percentage reduction of the average wound width and are expressed as the mean ± SEM of three independent experiments. **P<0.01, two-tailed t test.

## Results

### CD157 Expression Modulates Ovarian Cancer Cells Morphology and Cell-cell Interaction

We chose OVCAR-3 ovarian cancer cells as the most suitable model for studying the effects induced by CD157 expression since they have an epithelial phenotype [24], are poorly invasive, scarcely motile on plastic and barely anchorage-independent [25]. To explore the biological significance of CD157 in ovarian cancer progression, we stably transfected full length CD157 in CD157-negative OVCAR-3 cells (Figure 1A). Light microscopy images revealed that mock cells grew as tightly connected clusters composed of cells with typical cobblestone-like epithelial morphology. In contrast, OVCAR-3/CD157 cells exhibited a more scattered distribution and elongated shape, a distinctive feature of fibroblast-like cells, and formed poorly organized junctions between adjacent cells (Figure 1B,C). Consistently with this observation, phalloidin staining in OVCAR-3/CD157 cells revealed remodeling in actin cytoskeleton architecture which is a prerequisite for cancer cell motility and invasion [26], and is considered a characteristic of mesenchymal differentiation. Indeed, in OVCAR-3/CD157 cells, cortical actin, which is prevalent in mock cells, was largely replaced by the formation of F-actin stress fibers throughout the cells and accumulation at the adhesion sites, typical features of a cell spreading response (Figure 1D). The morphological features along with the reorganization of F-actin suggest that exogenous expression of CD157 in OVCAR-3 cells drives tumor cells toward morphological changes reminiscent of mesenchymal-like differentiation.

The formation of organized intercellular contacts critically affects the growth pattern of EOC cells and, in particular, may counteract their dissociation from the tumor mass, thus limiting the peritoneal dissemination of the tumor. To determine the effects of CD157 on the propensity of tumor cells to form aggregates under anchorage-independent conditions, an *in vitro* situation mimicking the early stages of the metastatic process occurring *in vivo*, we measured the formation of cell aggregates under slow agitation. The results showed that OVCAR-3/CD157 cells formed significantly fewer clusters than mock cells (P<0.01) (Figure 1E). The impaired ability of CD157-positive cells to form large, organized clusters in the absence of adhesion with a substrate was confirmed by a cell aggregation assay. In these experimental conditions, OVCAR-3/mock cells generated a huge number of compact and large clusters able to resist mechanical disruption, while OVCAR-3/CD157 cells formed small and scarcely cohesive clusters, apt to be destroyed (Figure 1F).

**Figure 5:**
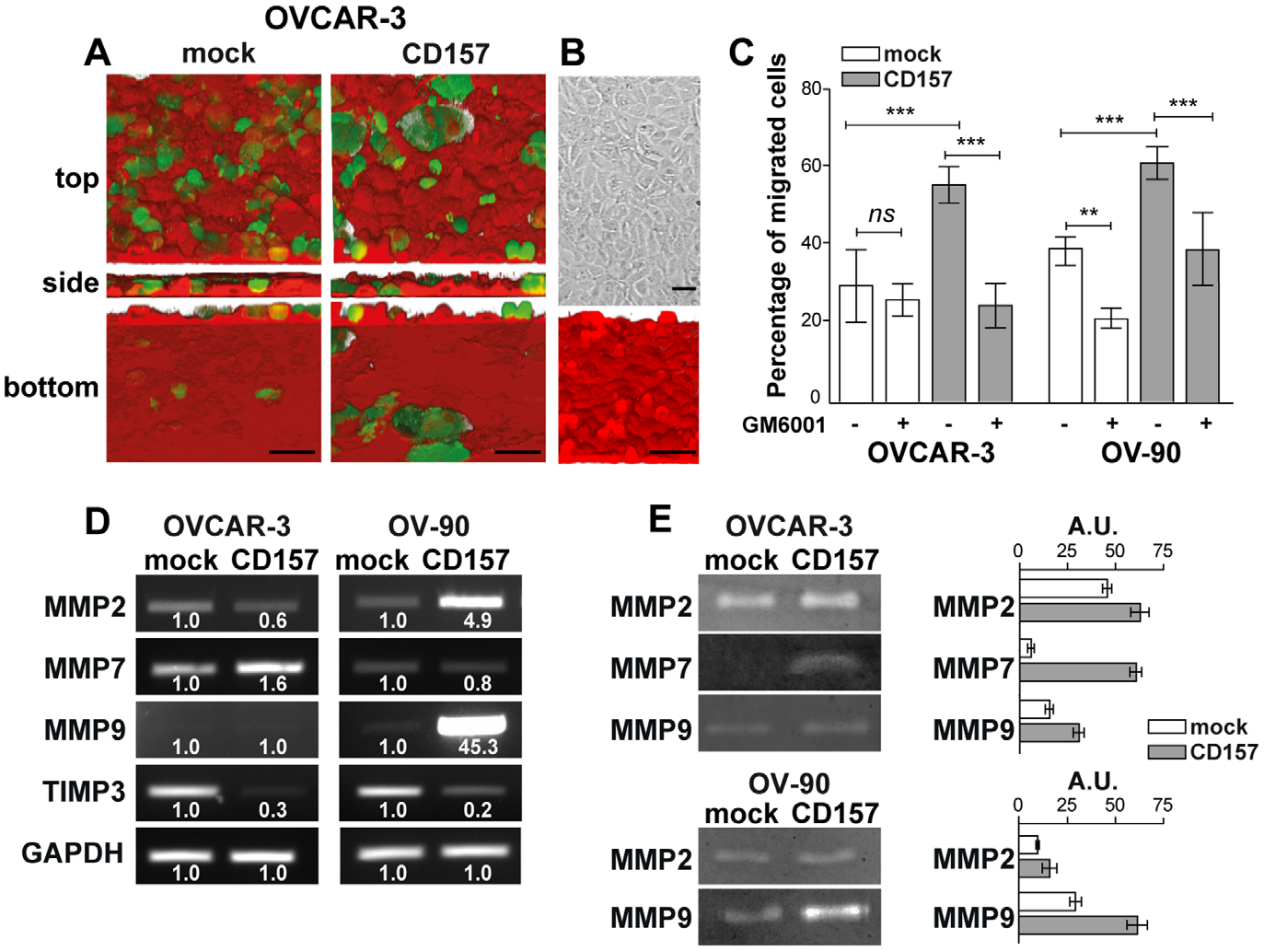
CD157 overexpression promotes invasion of mesothelium by OVCAR-3 and OV-90 cells and increases matrix metalloproteinase expression and activity. (A) Ovarian cancer cell migration through a mesothelial monolayer. Met-5A mesothelial cells were labeled with CellBrite™ Red and grown to confluence on fibronectin-coated coverslips. CFSE-stained tumor cells were plated onto the monolayer. After 6 h (OVCAR-3 cells) or 2.5 h (OV-90 cells) at 37°C, sample were fixed and analyzed using an Olympus FV300 laser scanning confocal microscope by sequential scanning of the XY planes recorded along the Z-axis (step size: 1.25 µM). Series of confocal optical XY images were processed using a 3D reconstruction program (bioView3D software, Bio-Image Informatics, University of California, Santa Barbara, CA). Top, orthogonal and bottom views are shown (scale bar: 50 µM). (B) Mesothelial layer integrity was verified by confocal microscopy analysis in CellBrite™ Red-labeled samples. Samples were analyzed with an Olympus FV300 laser scanning confocal microscope and by Nomarski differential interference contrast (DIC) optics. Scale bars : 50 µM. (C) Transmesothelial migration of OVCAR-3 and OV-90 cells. Where indicated, OVCAR-3/CD157, OV-90/CD157 and the corresponding mock cells were treated for 1 h with GM6001 (25 µg/ml) before seeding onto Met-5A mesothelial cell monolayer. Results are expressed in terms of percent of transmigrated cells calculated as the ratio of cells that crossed the mesothelial layer to the total number of cells in the field. At least ten fields have been counted for each sample. Results are expressed as the mean ± SEM of three independent experiments. P values were derived from analysis of variance (ANOVA) with Dunnett correction. **P<0.01, ***P<0.001, *ns,* not significant. (D) sqRT-PCR expression of MMP2, MMP7, MMP9 and TIMP3 in engineered OVCAR-3 (left panel) and OV-90 cells (right panel). Densitometry quantifies the levels of expression of MMP compared with GADPH. (E) Gelatin zymography and casein zymography were used to quantify MMP2, MMP9 and MMP7 activity, respectively, in cell-free conditioned media from OVCAR-3/CD157, OV-90/CD157 and the corresponding mock cells (left panel). Zymographic bands from all samples were quantified by densitometry (right panel). The enzyme activities are expressed as arbitrary units. Results are from one representative experiment performed in triplicate and are expressed as the mean ± SD.

In patients, cell detachment from the primary tumor and loss of contact with the ECM cause anoikis (detachment-induced apoptosis) in a large proportion of cells; only a reduced number of tumor cells acquire the ability to avoid anoikis and subsequently form invasive foci. Consistently, the expression of anti-apoptotic molecules, which confer resistance to anoikis, has been shown to promote metastasis in selected experimental models [27]. The observed association between high CD157 expression and tumor relapse in patients fostered the hypothesis that CD157 might provide protection against anoikis. As shown in Figure 1G, after 72 h in suspension, OVCAR-3/CD157 cells appeared as small aggregates or single cells, whereas control cells were organized in large, floating clusters. Comparison of anoikis sensitivity of OVCAR-3/CD157 and mock cells highlighted that the former had a significantly lower number of apoptotic cells than OVCAR-3/mock cells, at any of the times considered (Figure 1H). Cell cycle analysis confirmed that, after 48 h under anchorage-independent conditions, OVCAR-3/CD157 cells included a larger fraction of cycling cells compared to the control (Figure 1I). Next, we investigated whether the increased resistance to anoikis might influence the ability of OVCAR-3/CD157 cells to form colonies in soft agar, an *in vitro* conventional measure of tumorigenicity. Results in Figure 1J show that OVCAR-3/CD157 cells formed a greater number of colonies than OVCAR-3/mock cells. These data indicate that the ectopic expression of CD157 in OVCAR-3 cells is critical for cell cohesion, protects floating cells from anoikis and enhances *in vitro* tumorigenicity.

**Figure 6:**
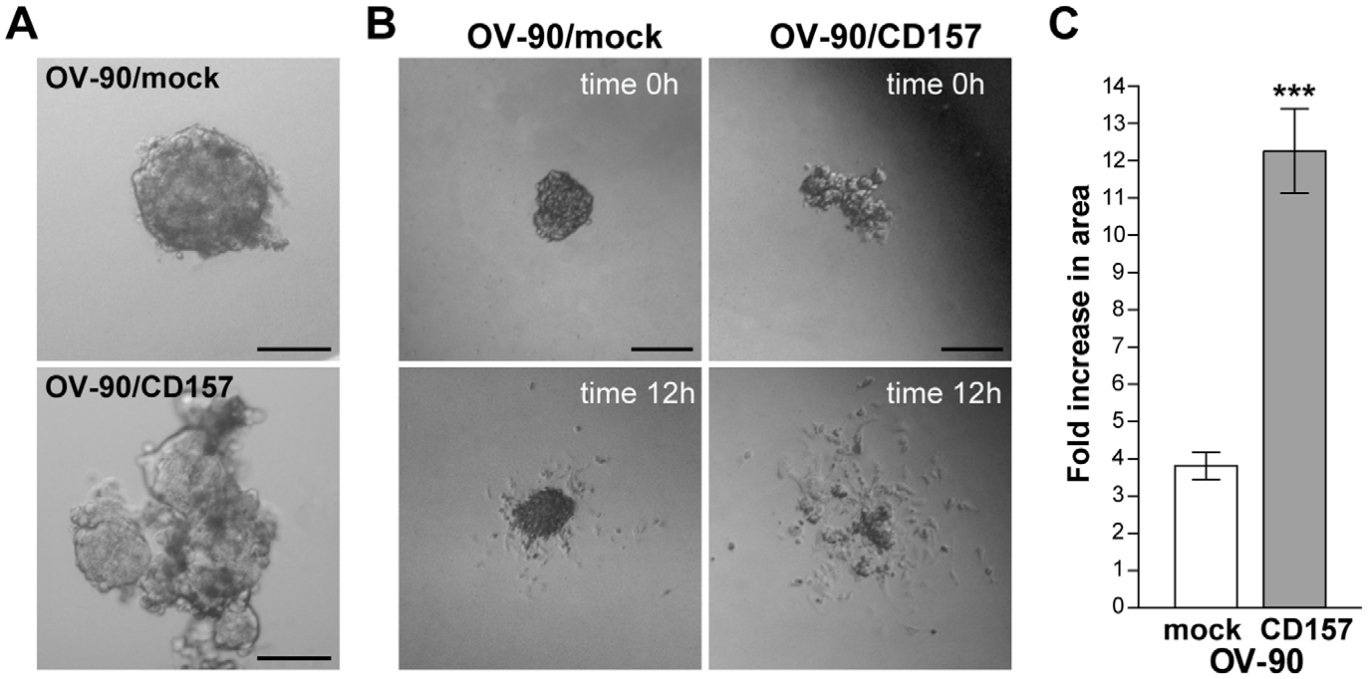
High CD157 expression influences spheroid formation and disaggregation in OV-90 cells. (A) Representative morphology of spheroids formed by OV-90/mock (top) and OV-90/CD157 (bottom) (scale bar: 100 µM). (B) Phase contrast microscopy images of spheroid disaggregation on fibronectin. A representative spheroid of OV-90/mock and OV-90/CD157 cells at time 0 and at 12 h are shown (scale bar: 200 µM). (C) Spheroids were photographed at time 0 and at 12 h, and the increase in the size of disaggregation area calculated as described in Materials and Methods is reported. The means ± SD of the fold change in area of 20 spheroids per condition of a representative experiment, repeated (n = 3), are shown. ***P<0.001, two-tailed t test.

### CD157 Regulates Epithelial and Mesenchymal Protein Markers

The accepted paradigm of oncogenic mesenchymal differentiation is that tumor cells reduce or lose their markers of epithelial cells and express *de novo* mesenchymal markers. Numerous reports have shown that this change in phenotype impairs cell-cell adhesion and communication and assists the dissemination of the tumor [28]–[30]. We therefore explored the relationship between the observed CD157-induced morphological alterations and mesenchymal differentiation of OVCAR-3 cells. Confocal microscopy analysis revealed that E-cadherin staining (a prototypic epithelial marker) was significantly lower in CD157-positive cells than in OVCAR-3/mock cells (Figure 2A). The cytoplasmic domain of E-cadherin has been shown to bind to the cytosolic protein β-catenin, which in turn provides anchorage to the actin cytoskeleton [31]. When E-cadherin is repressed, β-catenin is released and re-localizes in the cytoplasm before being targeted for degradation or translocating to the nucleus. As expected, β-catenin expression was lower in OVCAR-3/CD157 cells than in the control, and the weak residual staining was confined to the cytoplasm (Figure 2B, right panel) and, at least in part, it was localized into the nucleus (Figure 2B, inset). In OVCAR-3/mock cells E-cadherin and β-catenin staining was predominantly associated with inter-epithelial junctions (Figure 2A,B left panels). Western blotting of whole cell lysates confirmed the decreased expression of both E-cadherin and β-catenin in OVCAR-3/CD157 cells (Figure 2C). Cell fractionation and subsequent analysis of the cytoplasmic and nuclear fractions showed nuclear accumulation of β-catenin in OVCAR-3/CD157 cells (Figure 2D). SqRT-PCR revealed that the reduction of E-cadherin observed in OVCAR-3/CD157 cells was accompanied by increased expression of N-cadherin transcript (Figure 2E). However, the induction of N-cadherin was not appreciable at protein level (data not shown). The observed CD157-induced effects were not limited to a single ovarian cancer cell line, indeed, transient transfection of CD157 in CD157-negative TOV-21G epithelial cells (Figure S1A) was accompanied by decreased expression of E-cadherin and slightly increased expression of N-cadherin proteins (Figure S1B), and caused accumulation of β-catenin in the nuclear extracts (Figure S1C). Moreover, in A2780 cells (which lack expression of E-cadherin and present a mixed epithelial/mesenchymal phenotype [32]), exogenous expression of CD157 (Figure S1A) enhanced the basal level of N-cadherin, driving cells toward a mesenchymal differentiation (Figure S1B). These results indicate that morphological and phenotypic changes induced by ectopic expression of CD157 in ovarian cancer cells are consistent with their mesenchymal differentiation.

### CD157 Modulates the Expression of Transcription Repressors of E-cadherin

Reduced expression of E-cadherin can be achieved in multiple ways, among which, transcriptional repression has recently emerged as a fundamental mechanism for the dynamic silencing of the E-cadherin gene (*CDH1*) during tumor progression [3], [33]. Using sqRT-PCR and qRT-PCR, we determined whether CD157 expression was associated with increased transcription of some known repressors (such as Snail, Slug, Zeb1, Zeb2 and Twist1). Higher mRNA expression levels for Snail, Zeb1 and Twist1 (but not for Zeb2 and Slug) were observed in OVCAR-3/CD157 as compared to OVCAR-3/mock cells (Figure 2E). qRT-PCR confirmed that Snail and Zeb1 mRNA expression was increased by >50% in CD157-positive cells compared to control cells, while Twist1 mRNA expression did not significantly differ in CD157-positive versus negative cells (Figure 2F). Western blot analysis confirmed the increased expression of Snail and Zeb1 proteins in OVCAR-3/CD157 (Figure 2G), in TOV-21G/CD157 and in A2780/CD157 cells (Figure S1B) compared to the corresponding mock controls. These results suggest that ectopic expression of CD157 in ovarian cancer cells enhances the expression of Snail and Zeb1 transcriptional repressors, driving EMT.

**Figure 7:**
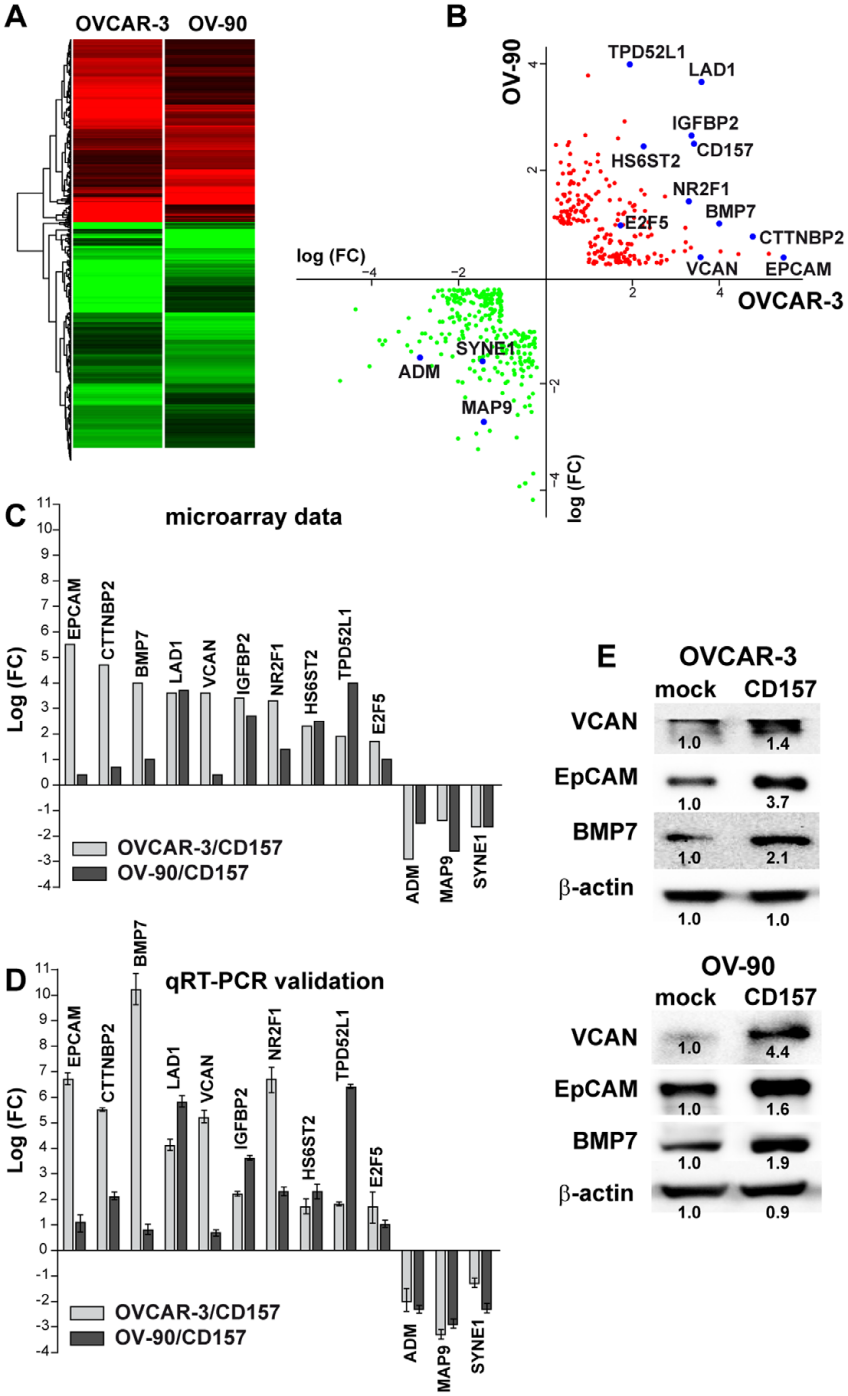
Gene expression profiling of OVCAR-3 and OV-90 cells overexpressing CD157. (A) Hierarchical clustering applied to the expression matrix of genes similarly regulated in both OVCAR-3 and OV-90 cells overexpressing CD157, using Euclidean distance as similarity metrics and complete linkage as the linkage method. A red-to-green gradient was used to indicate, for each gene, levels of up- or down-regulation. (B) Dot plot shows 378 significantly modulated genes (163 up-regulated and 215 down-regulated) shared by OVCAR-3/CD157 and OV-90/CD157 cells. Single genes are indicated by red (up) and green (down) data points. (C, D) A panel of modulated genes was selected and validated by qRT-PCR. (C) Fold changes of the various indicated genes in OVCAR-3 and OV-90 cells following CD157 overexpression are shown. (D) qRT-PCR validation of the genes shown in panel C. The comparative CT method was used to determine gene expression in CD157-transfected cells relative to the value observed in the mock-transfected cells, using TBP as normalization control. Histograms report the means ± SD of a qRT–PCR experiment conducted in triplicate. (E) The expression of VCAN, EpCAM and BMP7 was examined in OVCAR-3/CD157, OV-90/CD157 and the corresponding control cells by western blot analysis. Densitometry quantifies the expression level of the indicated proteins relative to β-actin. Results shown are representative of three independent experiments with similar results.

### CD157 Expression Enhances Motility and Invasion of Mesothelium by EOC Cells in vitro

Mesenchymal differentiation makes an essential contribution to cancer progression because it endows tumor cells with motile and invasive skills [29], [34]. Recently, we demonstrated that exogenous expression of CD157 in OVCAR-3 cells substantially increased cell motility [13] a prerequisite for cancer progression and for invasive migration of tumor cells into surrounding tissues. To evaluate the impact of increasing levels of CD157 on the behavior of tumor cells with an intermediate basal CD157 expression, we transfected the full-length CD157 in OV-90 EOC cells which are highly invasive and express low E-cadherin and CD157 (Figure 3A,C). OV-90/mock cells generated colonies with a typical epithelial shape, while OV-90/CD157 cells formed scattered colonies (Figure 3B), mirroring those formed by OVCAR-3/CD157 cells. Moreover, OV-90/CD157 displayed increased resistance to anoikis. The effect was appreciable after 48 h of anchorage-independent culture and persisted over time (Figure 3D). The increased viability of OV-90/CD157 cells under anchorage independent culture was paralleled by increased ability to form colonies in soft agar (Figure 3E). Moreover, OV-90/CD157 (similarly to OVCAR-3/CD157 cells [13]) exhibited a migratory potential two fold higher than the corresponding mock cells, as assessed using a wound healing assay (Figure 3F,G). Despite the correlation between expression levels of CD157 and tumor cell motility and tumorigenicity, OV-90 cells expressing high CD157 or basal CD157 showed no substantial differences in the E-cadherin and N-cadherin mRNA expression (Figure 3C). Next, to determine the contribution of endogenous CD157 expressed by OV-90 cells in tumor cell behavior, OV-90 cells were retrovirally transduced with a shRNA that targets the CD157 mRNA (OV-90/shCD157), resulting in efficient knockdown of CD157 expression (Figure 4A). OV-90/shCD157 cells appeared even more compact and organized in tight colonies than the OV-90/scramble cells (Figure 4B). This morphological change was accompanied by upmodulation of E-cadherin and downmodulation of Snail, Twist and Slug gene transcription (Figure 4C). Moreover, OV-90/shCD157 cells displayed increased sensitivity to anoikis (Figure 4D), reduced ability to form colonies in soft agar (Figure 4E) and a striking impaired migratory ability compared to cells transduced with a control shRNA (Figure 4F,G), suggesting that endogenous CD157, although it is expressed at low level, influences OV-90 cell motility and tumorigenicity. These findings were confirmed in a second cell model. We transduced OC314 cells (expressing CD157 levels comparable to that of OV-90 cells) with two independent shRNA resulting in efficient (OC314/shCD157) and partial (OC314/shCD157#2) decrease in CD157 mRNA and protein expression, respectively (Figure S2A). OC314/shCD157 cells exhibited significantly reduced motility, while OC314/shCD157#2 cells showed only slightly reduced motility as compared to OC314/scramble cells. (Figure S2B,C). Overall, these results strongly support a physiological role of CD157 in migration of selected EOC cell lines and highlight a direct correlation between CD157 expression levels and the efficiency of tumor cell motility.

**Figure 8:**
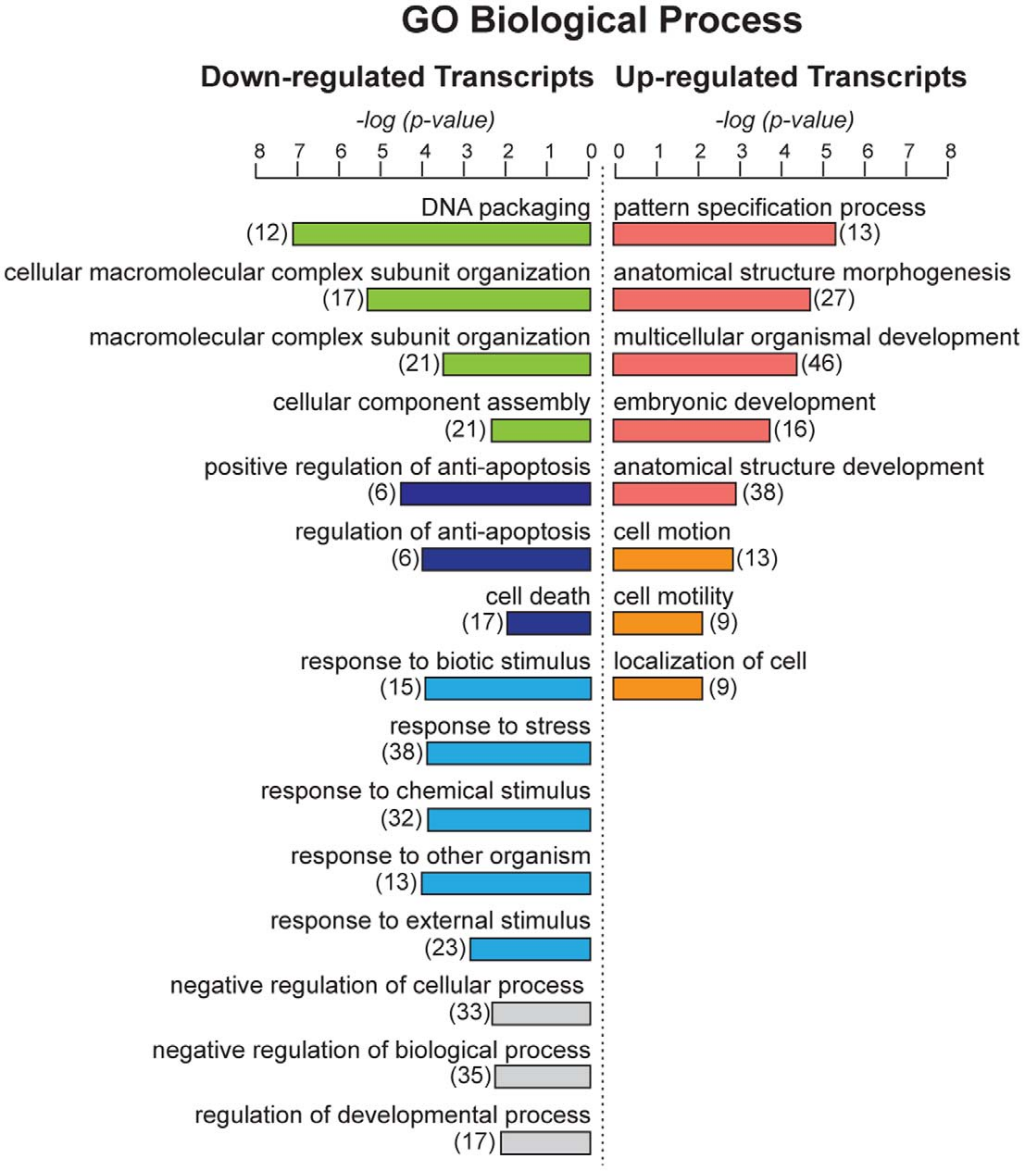
Gene ontology analysis of genes modulated by CD157 overexpression. Go analysis of differentially induced or repressed genes shared by OVCAR-3/CD157 and OV-90/CD157 cells with respect to enrichment of genes with assignments to specific biological processes. The number of genes in a specific biological process is indicated in brackets.

The adhesion of single cells (or cell aggregates) to and migration throughout the mesothelium are key steps during EOC metastatisation. The ability of CD157-positive and negative tumor cells to invade the mesothelium was compared using a specially designed 3D assay (Figure 5A,B). The evaluation of transmigration efficiency showed that forced expression of CD157 resulted in a significant increase in transmesothelial migration of both OVCAR-3 and OV-90 cells, as compared to the respective mock cells (Figure 5C), highlighting a pro-invasive function of CD157 in EOC cells. Collectively, these data indicate that CD157 expression impacts on tumorigenicity and invasiveness of ovarian cancer cells *in vitro*.

### CD157 Expression Enhances Matrix Metalloproteinase Expression and Activity

Tumor invasion is supported by active MMPs secreted by tumor or stromal cells, which constitute the most prominent family of proteinases associated with tumorigenesis [35]. Hence, following the observation that the higher the expression of CD157, the greater the ability of tumor cells to invade the mesothelium, we hypothesized that CD157 expression could promote MMP activity thus fuelling tumor cell transmigration. To address this issue, MMP expression was determined by measuring MMP mRNA and proteolytic activity. Forced expression of CD157 was characterized by increased expression of MMP2 and MMP7 in OVCAR-3 cells, and of MMP2 and MMP9 in OV-90 cells. Concomitantly, a remarkable reduction of the tissue inhibitor of metalloproteinases 3 (TIMP3) was appreciable in both cell lines (Figure 5D). The results of zymography assays showed an increased gelatino- or caseino-lytic activity in conditioned media derived from CD157-overexpressing cells (Figure 5E). The increased proteolytic activity significantly contributes to the enhanced trans-mesothelial migration of ovarian cancer cells with high levels of CD157. Indeed, in the presence of GM6001 (a broad-spectrum inhibitor of MMPs) both OVCAR-3/CD157 and OV-90/CD157 cells transmigrated with an efficiency comparable to that of the corresponding untreated mock cells. GM6001 also interfered with trans-mesothelial migration of the highly invasive OV-90/mock cells but not of the barely invasive OVCAR-3/mock cells (Figure 5C). These results reflect an intense proteolytic activity associated with CD157 overexpression in EOC cells which significantly contributes to increase the invasive potential characterizing cells expressing high CD157.

### CD157 Overexpression Influences Spheroid Formation and Disaggregation

The formation of cellular spheroids capable of floating in the abdominal cavity and overcoming the environmental stresses is an important step during tumor dissemination in EOC patients [36]. We generated spheroids from OV-90 cells and determined whether the CD157-induced phenotype can overthrow the spherical architecture acquired by OV-90 cells grown in suspension. Accordingly with their natural propensity to form spheroids [37], OV-90-mock cells gave rise to compact spherical clusters (Figure 6A, top panel); in contrast, OV-90/CD157 cells formed irregular clusters composed of loosely associated cells (Figure 6A, bottom panel). The disruption of the spherical architecture was accompanied by a considerable increase in the invasive properties of cells. Indeed, when these disorganized spheroids were seeded onto fibronectin-coated dishes a number of cells rapidly escaped from the cell aggregates and invaded a large area of the surrounding matrix, whereas, under similar conditions, OV-90/mock spheroids maintained their quite compact structure and cells leaking from these spheroids invaded a significantly smaller area than the OV-90/CD157 cells (Figure 6B, C). These results further support the association between high CD157 expression and the enhanced invasive proclivity of EOC cells.

### Gene Expression Profiling Identifies Molecular Changes Induced by the Overexpression of CD157 in OVCAR3 and OV-90 Cells

To dissect the transcriptional changes that may mediate the tumor aggressiveness associated with high CD157 expression, we performed microarray gene expression analysis of OVCAR-3 cells with and without CD157 and OV-90 cells with increased or basal expression of CD157. According to our selection criteria, 378 unique significantly modulated transcripts (163 upregulated and 215 downregulated) (Table S3 and S4), corresponding to 480 probes, were shared by OVCAR-3/CD157 and OV-90/CD157 cells (Figure 7A,B). To validate the microarray data, ten upregulated genes (namely, *EPCAM, CTTNBP2, BMP7, LAD1, VCAN, IGFBP2, NR2F1, HS6ST2, TPD52L1, E2F5*) and three down-regulated genes (namely *ADM, MAP9, SYNE1*) were chosen for validation by qRT-PCR based on their levels of expression (Figure 7B). The results of qRT-PCR confirmed those obtained by our microarray analysis, with minor differences in amplitude in the fold change expression (Figure 7C,D). Interestingly, among the validated genes, *EPCAM*, *VCAN, HS6ST2* and *TPD52L1*, whose expression was increased in CD157-overexpressing cells, proved to be decreased in OV-90/shCD157 cells (Figure S3). The modulation of selected genes such as BMP7, EpCAM and VCAN entailed an increase in the proteins encoded by them (Figure 7E), indicating that high CD157 expression alters tumor cell phenotype.

Functional grouping and assessment of the Gene Ontology (GO) designations of the 378 deregulated genes in CD157-transfected cells indicated that upregulated genes included a substantial number of genes known to be implicated in development, which also includes EMT, (such as *S100A4, BMP7, WNT10A, WNT6, FGF9, FZD4, FZD7, SFRP1, EPCAM* and ten homeobox *HOXB3, DLX1, TSHZ1, HOXB8, HOXB5, SIX1, ONECUT2, MNX1, HOXA10, HOXB9*, among others), as well as genes related to the control of cell motility and migration (for example, *VAV3, FUT8, PODXL, EFNB1, TPM1, SEMA6A, CTTNBP2, PVRL1, SIX1, VCAN, NR2F2, NR2F1*), and genes involved in the ECM-receptor interactions (such as *COL4A1, COL4A2, ITGAV, ITGB4, LAMC2*). The list of downregulated genes comprised genes playing a role in the control of protein-DNA complex assembly (such as *H1F0, HIST1H2AC, HIST2H2AA4, HIST1H2AD, HIST1H2AE, HIST1H3D, SIRT1, HIST1H4H*) and of apoptosis and cell death (such as *CASP5, TP53I3, IL6, CASP4, CASP9, ERBB4, DUSP1, CARD16, GULP1, BCL2A1, CASP1, PPP1R15A, EMP2*) (Figure 8 and Table S5).

GeneGo pathways and process networks analysis of genes representing the signature of CD157-overexpressing OVCAR-3 and OV-90 cells revealed a significant enrichment of genes belonging to selected cell adhesion and ECM/cytoskeleton remodeling signaling pathways, whose implication in tumor progression is well documented. Similar process networks were also on the top of the list. Moreover, networks with highest scores included Notch signaling, connective tissue degradation (associated for example, with upregulation of the metalloproteinase *ADAM15* and down-regulation of *TIMP3*), apoptosis (associated for example, with down-modulation of key elements of the caspase cascade and the regulation of the neuregulin/erbB pathway) and development, including regulation of EMT and blood vessel morphogenesis (Table S5). Collectively, these data indicate that forced expression of CD157 in EOC cells modulates the transcription of a spectrum of genes encoding proteins involved in crucial aspects of ovarian cancer dissemination.

## Discussion

In this study we addressed the relevance of CD157 in the induction of EOC aggressiveness and provided evidence that CD157 overexpression is associated with dramatic variations in tumor cell morphology, decreased cell-cell interactions, increased anchorage independent growth, motility, and mesothelial invasion. Indeed, ectopic expression of CD157 in OVCAR-3 cells, as well as its increased expression in OV-90 cells, resulted in reduced cell-cell contacts and adherens junction organization and enhanced cell spreading, improving the ability of these tumor cells to move and migrate as compared to the corresponding mock cells. The exogenous expression of CD157 proved to be sufficient to convert immobile OVCAR-3 epithelial cells into mesenchymal-like cells characterized by weak contacts between neighboring cells, increased motility, invasiveness, tumorigenicity, and improved resistance to anoikis, all properties known to be fundamental prerequisites for the progression of primary tumors to metastatic disease [38]. These findings suggest that CD157 may function as a potent driver of EOC progression. The observed association between high levels of CD157 and the likelihood of disease recurrence in patients with EOC implicitly supports a role of CD157 in the control of tumor progression also *in vivo* [13].

There is a growing consensus that the events that convert adherent and strictly connected tumor epithelial cells into migratory cells capable of invading the ECM and establishing distant metastases are reminiscent of the EMT occurring during development [39]. In the ovary, EMT is a physiological process during the postovulatory repair; in pathological contexts, such as in tumors, however, it may have a detrimental effect, promoting metastasis [29]. One of the leading events for EMT is the downregulation of E-cadherin expression and function, which is considered the hallmark of this process [30]. Despite the fact that primary EOC express E-cadherin, advanced tumors have reduced E-cadherin expression or none at all, suggesting that downregulation of E-cadherin is associated with the acquisition of the invasive phenotype by EOC cells [40]. Exogenous expression of CD157 in OVCAR-3 and TOV-21G cells that present typical epithelial features, proved to suppress E-cadherin and to enhance N-cadherin expression with consequent intracellular relocation and partial nuclear translocation of β-catenin. In A2780 cells that present a mixed epithelial/mesenchymal phenotype, forced expression of CD157 increased the basal level of N-cadherin and other mesenchymal traits. The CD157-driven differentiation toward a mesenchymal phenotype is mainly choreographed by Snail and Zeb1 transcriptional repressors. It is now evident that, beside inducing the EMT program, these transcriptional factors also confer resistance to apoptosis [33]. Furthermore, ZEB1 can contribute to stemness maintenance thus enhancing the ability of tumor cells to both disseminate and to fuel the growth of metastases [41].

A crucial step in the progression of EOC is the release of tumor cells into the peritoneal cavity. Once detached from the original site, tumor cells disseminating throughout the peritoneum lose their attachment to the neighboring cells and to the ECM, resulting in anoikis [42]. We found that exogenous expression of CD157 rescues tumor cells from anoikis despite it reduces the ability of cells to form large aggregates, which are considered a defense of tumor cells from anoikis. This apparent inconsistency with what is thought to be the rule in ovarian cancer, suggests that i) small aggregates of cells provide more favorable conditions for anchorage-independent tumor cell survival than large aggregates, and that ii) tumor cells expressing high CD157 likely develop strategies relying on specific pro-survival signals, allowing both individual cells and small aggregates that have detached from the tumor to escape anoikis and to grow under anchorage-independent conditions. The identification of the molecular basis of these signals is expected to shed light on this issue. This observation leads us to assume that in patients with advanced ovarian cancer, high expression of CD157 may confer resistance to cell death induced by the loss of adhesive supports, thus generating a subpopulation of viable, highly malignant cells that might account for a rapid tumor relapse.

Mesenchymal differentiation of EOC cells implies increased secretion of MMPs which degrade and remodel the ECM, paving the way to the establishment of metastases and the sprouting of new vessels. Apart from this conventional activity, MMPs are emerging as key modulators of the tumor microenvironment contributing to the formation of a metastatic niche [43]. Our data show that CD157 regulates secretion of tumor-specific MMPs, such as MMP2 and MMP7 (in OVCAR-3 cells) which play a major role in early metastasis [44] and MMP9 (in OV-90 cells), which is implicated in matrix invasion [45], is elevated in invasive ovarian cancer specimens as well as ovarian carcinomatous ascites, and correlates with lymph node metastasis [46]. The significant reduction of TIMP3 (an endogenous inhibitor of MMPs) and increase of ADAM15 transcripts, shared by both cell lines, emphasize an imbalance of MMPs functions in tumor cells overexpressing CD157.

In ovarian cancer, proteolytic degradation of ECM assists the release of cells from the tumor mass and allows them to anchor to and invade through the mesothelium establishing secondary lesions and, at a later stage, to metastasize to distant organs. Using a co-culture system to model aspects of the metastatic process occurring *in vivo* [17], we demonstrated that the extent of transmesothelial migration achieved by each EOC cell line correlates with the level of CD157 and is influenced by the activation of MMPs, further implicating CD157 in the control of a crucial step of ovarian cancer metastasis, that is, mesothelial invasion. We observed that the overexpression of CD157 in OV-90 cells (showing a naturally low level of CD157) was able to further increase their constitutive motility, reciprocally CD157 gene silencing reduced their basal motility, and that the extent of CD157 silencing correlated with motility in OC314 cells. Collectively, these data provide clear experimental evidence of a relationship between CD157 levels and the progression of metastatic ovarian cancer and support a direct role of CD157 in regulating the aggressiveness of EOC cells reinforcing our previous observation that high expression of CD157 is associated with adverse clinical outcome in patients [13].

To gain further insight into CD157 function in EOC progression, we investigated gene expression changes following CD157 transfection in OVCAR-3 and OV-90 cells. According to our *in vitro* results, the analysis of genes deregulated in each line confirmed the acquisition of clear mesenchymal traits in OVCAR-3/CD157 cells (including downregulation of *CDH1*) but not in OV-90/CD157 cells in which epithelial and mesenchymal traits coexist (GEO database, ID: GSE36364). The analysis of genes showing concordant modulation in both cell lines led to the identification of 378 significantly altered genes, representing the signature of both OVCAR-3 and OV-90 cells overexpressing CD157. The overall picture inferred from the analysis of these genes indicated that high CD157 expression results in strengthening of biological functions that favor tumor progression (for example, cell differentiation, cell motility and migration), and weakening of selected biological processes that hinder tumor progression, such as apoptosis, cell death and response to stress. Although the analysis of transcriptomic profiles alone is not enough to permit definitive conclusions on the overall effects of CD157-mediated EOC aggressiveness, however, its consistency with the experimental data and clinical observations strongly support the view that CD157 is directly implicated in the control of ovarian cancer progression. The coexpression of molecules such as BMP7, VCAN and EpCAM in EOC cells expressing high CD157 further substantiates this view since these molecules are considered negative prognostic markers involved in the control of the progression of various types of tumor. For instance, BMP7 overexpression has been implicated in EMT in prostate cancer [47] and with increased cell migration and invasion in breast cancer [48]. VCAN is a mesenchymal marker whose increased expression in ovarian cancer correspond to tumor progression, metastatic dissemination and poorer survival outcome [49], [50]. EpCAM is considered both an epithelial marker and ovarian cancer stem-like cells marker whose expression is associated with poor prognosis in ovarian cancer patients [51], [52]. By virtue of this double role attributed to EpCAM, the observed correlation between CD157 and EpCAM expression in EOC cells does not contradict the ability of CD157 to promote a mesenchymal-like phenotype. Indeed, emerging evidence suggests that acquisition of EMT and induction of cancer stem-like cell properties are interrelated and contribute to tumor recurrence and metastatic growth in several tumors [53]. The connection between EMT and stemness could confer a crucial advantage to a single tumor cell by increasing its ability to both disseminate and to begin a stemness-associated growth and differentiation program in the metastasis. The hypothesis that CD157 serves as a point of convergence in conferring mesenchymal and stem cell-like differentiation to ovarian cancer cells is very intriguing but it is beyond the scope of this study.

Although the results of this study were derived from a limited number of cell lines, the identification of a set of deregulated genes in EOC with high CD157 strongly suggests a list of candidate genes for further validation and functional analysis in patients with EOC.

In summary, these data provide mechanistic support to our previous studies [13], and demonstrate that the functional contribution of CD157 to EOC progression relies on its ability to switch on a differentiation program that allows cancer cells to overlook the rules of epithelial tissue architecture and to advance in their malignant progression. At the moment we don’t know whether CD157 regulates EOC progression and aggressiveness per se, or as part of a multimolecular complex governing cell motility during metastatic progression. However, since CD157 is devoid of transmembrane and cytoplasmic domain, and on the basis of our previous results [9], we hypothesize that CD157 may cooperate with other transmembrane receptors to fulfill its functions. Lateral partners of the CD157 interactome and mechanisms regulating CD157 interactions in ovarian cancer are currently under investigation in our lab.

Additional studies are needed to further validate the tumorigenic potential of OVCAR-3/CD157 and OV-90/CD157 cells in animal models; however, the *in vitro* data demonstrate that high expression of CD157 is sufficient to increase tumor aggressiveness and tumorigenicity in several epithelial ovarian cancer cell lines. It is tempting to speculate that CD157 might be a promising therapeutic target for therapies aimed at controlling the invasion and dissemination of the peritoneal cavity by ovarian cancer cells.

## Supporting Information

Figure S1 **CD157 overexpression alters the expression of epithelial and mesenchymal markers in TOV-21G and A2780 ovarian cancer cells.** (A) sqRT-PCR and western blot analysis of CD157 in mock- or CD157-transfected TOV-21G and A2780 cells. The anti-β-actin mAb and GAPDH were used as internal controls. (B) Western blot analysis of E-cadherin, β-catenin, N-cadherin EMT markers, and Zeb-1 and Snail transcription factors in total extracts from vector- or CD157-transfected TOV-21G and A2780 cells. Densitometry quantifies the expression level of the indicated proteins relative to β-actin. (C) β-catenin protein level in cytoplasmic and nuclear fractions of TOV-21G/mock and TOV-21G/CD157 cells were determined by western blot analysis. α-tubulin and lamin B1 were used as cytoplasmic and nuclear loading controls, respectively. Results shown are from a representative experiment repeated at least twice with similar results.
(TIF)

Figure S2 **Effects of CD157 knockdown in OC314 cells.** (A) sqRT-PCR and western blot analysis showing OC314 cells retrovirally transduced with two independent shRNA that targets the human CD157 mRNA, resulting in efficient and partial reduction of CD157 expression, respectively. GAPDH is shown as the internal control. (B) Effect of CD157 knockdown on OC314 cell migration in a scratch-wound assay. (C) The ability of OC314/scramble, OC314/shCD157 and OC314/shCD157#2 cells to close the wound was calculated by measuring 20 randomly chosen distances along the wound edge at time 0 and at 24 h. Results represent the percentage reduction of the average wound width and are expressed as the mean ± SEM of three independent experiments. **P<0.01, two-tailed t test.
(TIF)

Figure S3 **qRT-PCR of selected genes in OV-90/CD157 and OV-90/shCD157 cells.** Data show the log(FC) in OV-90/CD157 and OV-90/shCD157 cells of EPCAM, VCAN, HS6ST2 and TPD52L1 genes whose expression was increased in OV-90/CD157 cells and reduced in OV-90/shCD157 cells. The comparative CT method was used to determine gene expression in CD157-transfected or knock-down cells relative to the value observed in the corresponding control cells using TBP as normalization control. Histograms report the means ± SD of a qRT–PCR experiment conducted in triplicate.
(TIF)

Table S1 **Primers used for sqRT-PCR.**
(DOC)

Table S2 **Primers used for qRT-PCR.**
(DOC)

Table S3 **Genes up-regulated in OVCAR-3 and OV-90 cells overexpressing CD157** ***vs*** **the corresponding mock cells.**
(DOCX)

Table S4 **Genes down-regulated in OVCAR-3 and OV-90 cells overexpressing CD157** ***vs*** **the corresponding control cells.**
(DOCX)

Table S5 **GeneGo pathway maps and GeneGo process networks analysis of the CD157-associated molecular signature.**
(DOCX)
