# Supplementary figures and images for: Overexpression of CD157 Contributes to Epithelial Ovarian Cancer Progression by Promoting Mesenchymal Differentiation
Source: PLoS One. 2012 Aug 20;7(8):e43649. doi: 10.1371/journal.pone.0043649 (PMC3423388; doi:10.1371/journal.pone.0043649)

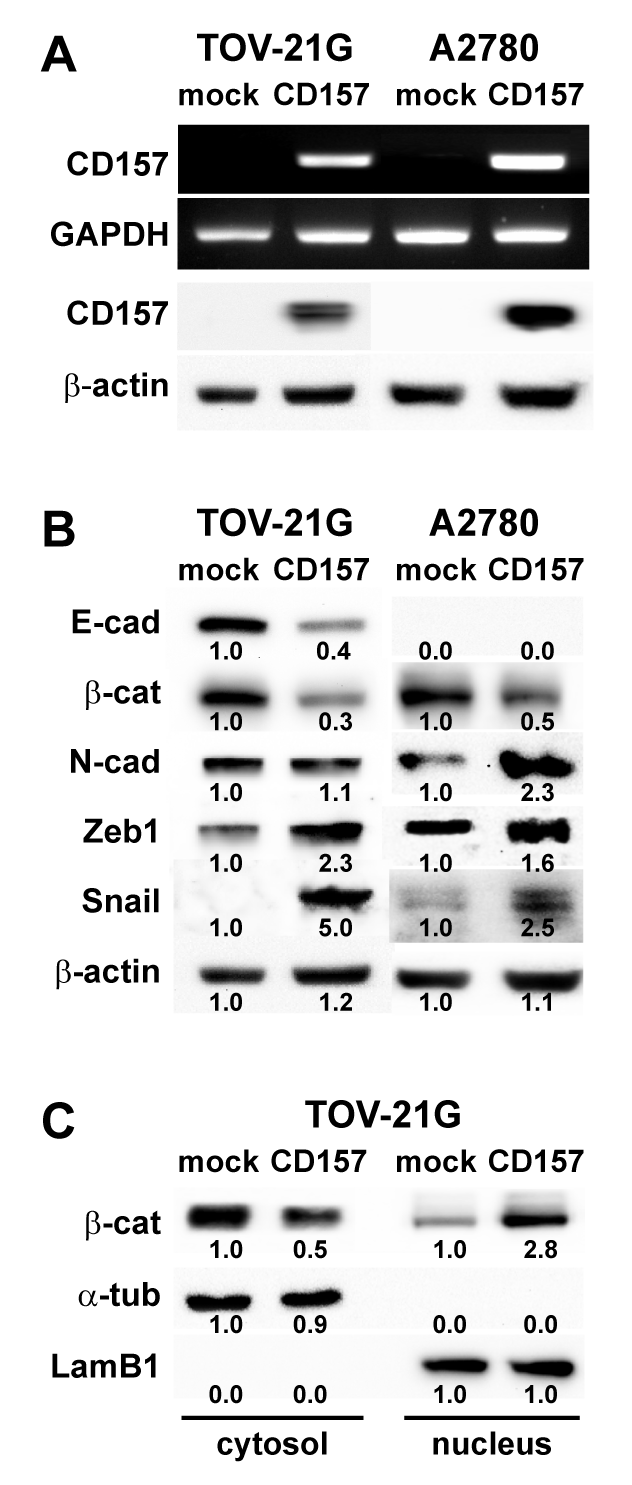

Supplement: Figure S1 — CD157 overexpression alters the expression of epithelial and mesenchymal markers in TOV-21G and A2780 ovarian cancer cells. (A) sqRT-PCR and western blot analysis of CD157 in mock- or CD157-transfected TOV-21G and A2780 cells. The anti-β-actin mAb and GAPDH were used as internal controls. (B) Western blot analysis of E-cadherin, β-catenin, N-cadherin EMT markers, and Zeb-1 and Snail transcription factors in total extracts from vector- or CD157-transfected TOV-21G and A2780 cells. Densitometry quantifies the expression level of the indicated proteins relative to β-actin. (C) β-catenin protein level in cytoplasmic and nuclear fractions of TOV-21G/mock and TOV-21G/CD157 cells were determined by western blot analysis. α-tubulin and lamin B1 were used as cytoplasmic and nuclear loading controls, respectively. Results shown are from a representative experiment repeated at least twice with similar results. (TIF) [file pone.0043649.s001.tif]

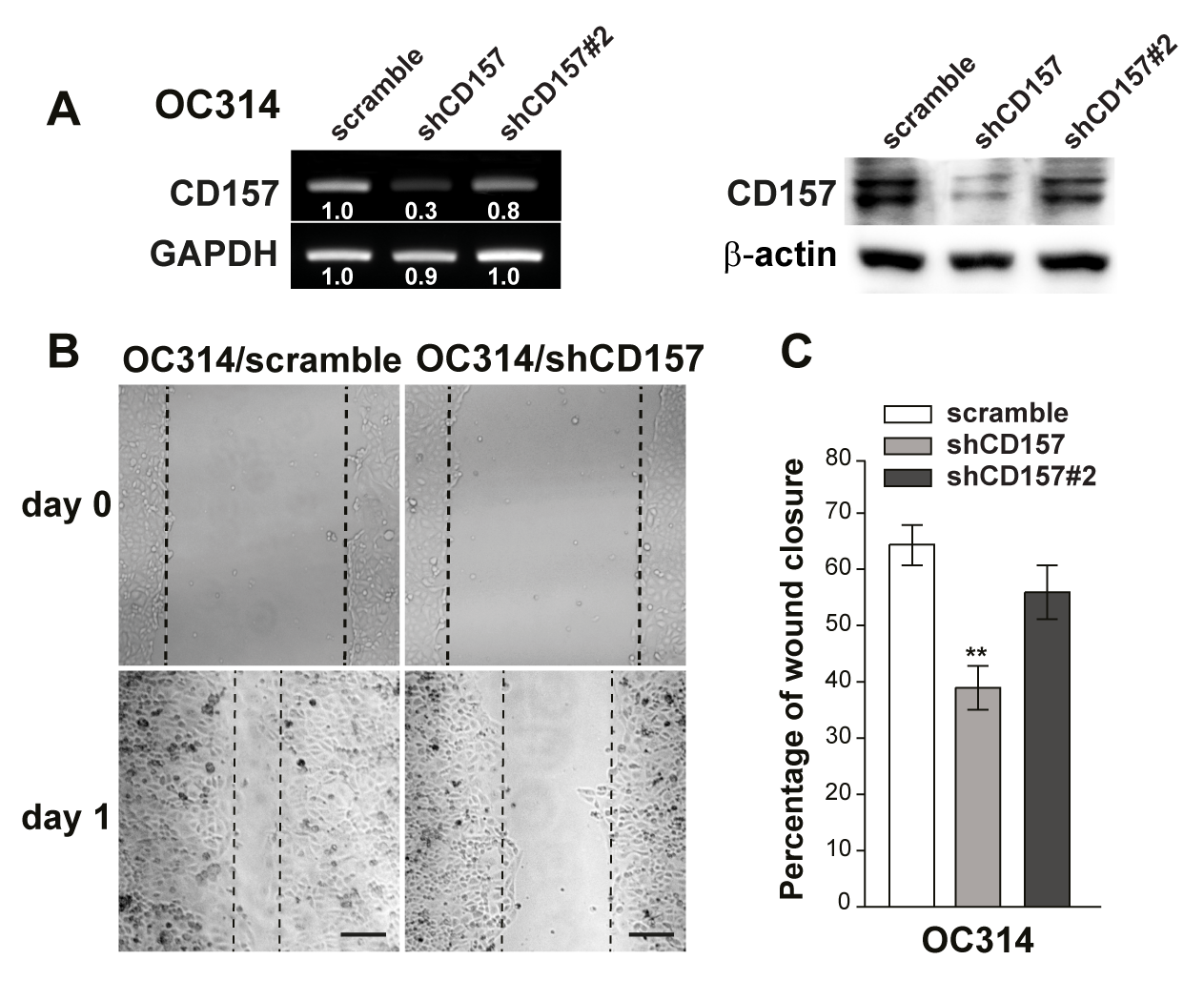

Supplement: Figure S2 — Effects of CD157 knockdown in OC314 cells. (A) sqRT-PCR and western blot analysis showing OC314 cells retrovirally transduced with two independent shRNA that targets the human CD157 mRNA, resulting in efficient and partial reduction of CD157 expression, respectively. GAPDH is shown as the internal control. (B) Effect of CD157 knockdown on OC314 cell migration in a scratch-wound assay. (C) The ability of OC314/scramble, OC314/shCD157 and OC314/shCD157#2 cells to close the wound was calculated by measuring 20 randomly chosen distances along the wound edge at time 0 and at 24 h. Results represent the percentage reduction of the average wound width and are expressed as the mean ± SEM of three independent experiments. **P<0.01, two-tailed t test. (TIF) [file pone.0043649.s002.tif]

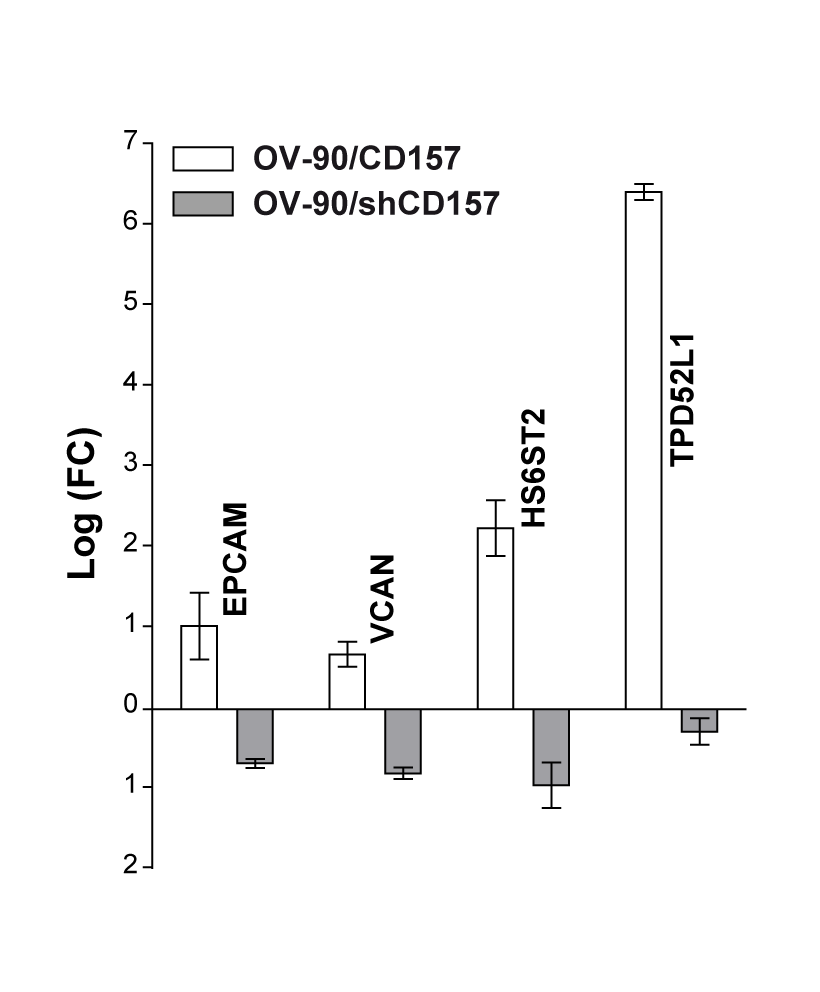

Supplement: Figure S3 — qRT-PCR of selected genes in OV-90/CD157 and OV-90/shCD157 cells. Data show the log(FC) in OV-90/CD157 and OV-90/shCD157 cells of EPCAM, VCAN, HS6ST2 and TPD52L1 genes whose expression was increased in OV-90/CD157 cells and reduced in OV-90/shCD157 cells. The comparative CT method was used to determine gene expression in CD157-transfected or knock-down cells relative to the value observed in the corresponding control cells using TBP as normalization control. Histograms report the means ± SD of a qRT–PCR experiment conducted in triplicate. (TIF) [file pone.0043649.s003.tif]
