# Supplementary material for: Overexpression of CD157 Contributes to Epithelial Ovarian Cancer Progression by Promoting Mesenchymal Differentiation
Source: PLoS One. 2012 Aug 20;7(8):e43649. doi: 10.1371/journal.pone.0043649 (PMC3423388; doi:10.1371/journal.pone.0043649)
Supplement: Table S1 — Primers used for sqRT-PCR. (DOC) [file pone.0043649.s004.doc]

**Table S1. Primers used for sqRT-PCR**

| **Oligo name** | **Forward primer**  **5'-3' sequence** | **Reverse primer**  **5'-3' sequence** | **Product size** | **Annealing**  **temperature** |
| --- | --- | --- | --- | --- |
| **CD157** | ACACTTGCGGGACATCTTCC | GGGAATAGAGTGCCTGGACA | 184 bp | <57°C> |
| **E-cadherin** | TGGAGGAATTCTTGCTTTGC | CGTACATGTCAGCCAGCTTC | 488 bp | <55°C> |
| **N-cadherin** | CACTGCTCAGGACCCAGAT | TAAGCCGAGTGATGGTCC | 416 bp | <55°C> |
| **β-catenin** | CCAGCGTGGACAATGGCTAC | CTCTGAGCTCGAGTCATTGC | 290 bp | 55°C |
| **Snail** | TTCCAGCAGCCCTACGACCAG | CGGACTCTTGGTGCTTGTGGA | 682 bp | 55°C |
| **Slug** | AAGCATTTCAACGCCTCCAA | AAGGTAATGTGTGGGTCCGA | 529 bp | 55°C |
| **Zeb1** | AGCAGTGAAAGAGAAGGG | GGTCCTCTTCAGGTGCCT | 229 bp | 55°C |
| **Zeb2** | GCGGCATATGGTGACACA | TGCCACTAAACCCGTGTGTA | 464 bp | 55°C |
| **Twist1** | GGAGTCCGCAGTCTTACGAG | TCTGGAGGACCTGGTAGAGG | 201 bp | 55°C |
| **MMP2** | GGCCCTGTCACTCCTGAGAT | GGCATCCAGGTTATGGGGGA | 474 bp | 55°C |
| **MMP7** | GGTCACCTACAGGATCGTATCATAT | CATCACTGCATTAGGATCAGAGAAA | 373 bp | 55°C |
| **MMP9** | GATGCGTGGAGAGTCGAAAT | CACCAAACTGGATGACGATG | 338 bp | 55°C |
| **TIMP3** | CTGACAGGTCGCGTCTATGA | TGTGGCATTGATGATGCTTT | 318 bp | 55°C |
| **GAPDH** | GAGTCAACGGATTTGGTCGT | TTGATTTTGGAGGGATCTCG | 238 bp | 55°C |
