# Supplementary material for: Overexpression of CD157 Contributes to Epithelial Ovarian Cancer Progression by Promoting Mesenchymal Differentiation
Source: PLoS One. 2012 Aug 20;7(8):e43649. doi: 10.1371/journal.pone.0043649 (PMC3423388; doi:10.1371/journal.pone.0043649)
Supplement: Table S2 — Primers used for qRT-PCR. (DOC) [file pone.0043649.s005.doc]

**Table S2. Primers used for qRT-PCR.**

| **Oligo name** | **Forward primer**  **5'-3' sequence** | **Reverse primer**  **5'-3' sequenze** | **Product size** |
| --- | --- | --- | --- |
| **ZEB1** | AACTGCTGGGAGGATGACAC | TCCTGCTTCATCTGCCTGA | 75 bp |
| **SNAIL** | GCTGCAGGACTCTAATCCAGA | ATCTCCGGAGGTGGGATG | 84 bp |
| **TWIST1** | CCCAACTCCCAGACACCTC | CAAAAAGAAAGCGCCCAAC | 96 bp |
| **EPCAM** | CGCAGCTCAGGAAGAATGTG | TGAAGTACACTGGCATTGACG | 88 bp |
| **CTTNBP2** | GAGAATGCCAGTGTCACAAAAG | TGTTGCTGCATTCAGTAGTTTG | 68 bp |
| **BMP7** | ACGCTTCGACAATGAGACG | TGTCGAGCAGGAAGAGATCC | 89 bp |
| **LAD1** | GCCACCTCTTTGAGAAGGAA | TTGATGTCACAACCCCTGAG | 96 bp |
| **VCAN** | GCACCTGTGTGCCAGGATA | CAGGGATTAGAGTGACATTCATCA | 70 bp |
| **IGFBP2** | GGTGGCAAGCATCACCTT | ACCTGGTCCAGTTCCTGTTG | 89 bp |
| **NR2F1** | ATCGTGCTGTTCACGTCAGA | GCTCCTCACGTACTCCTCCA | 102 bp |
| **HS6ST2** | AGACCCGGAACACATCTAAGAG | TCAAGTACCGGGACACTGG | 78 bp |
| **TPD52L1** | CATCTGCTCTGGGAAGCAC | GCAACGGTTCAGTCTCCAAC | 101 bp |
| **E2F5** | GCAACATGTCTCTGAAAGAAGC | GATATCTCCACTAATAGATCCTGCTGA | 85 bp |
| **ADM** | GCCTGCCCAGACCCTTAT | GTAGCGCTTGACTCGGATG | 100 bp |
| **MAP9** | CCTCTTATTTATCTCCTTACTCCTCCA | GCTTTGATCTGGTGGACAGTT | 61 bp |
| **SYNE1** | CCCCAAACAGACAGAAAACG | CTATGTGGACTGCTGACAGAGG | 76 bp |
| **TBP** | CGGCTGTTTAACTTCGCTTC | CACACGCCAAGAAACAGTGA | 75 bp |
