# Supplementary material for: Overexpression of CD157 Contributes to Epithelial Ovarian Cancer Progression by Promoting Mesenchymal Differentiation
Source: PLoS One. 2012 Aug 20;7(8):e43649. doi: 10.1371/journal.pone.0043649 (PMC3423388; doi:10.1371/journal.pone.0043649)
Supplement: Table S3 — Genes up-regulated in OVCAR-3 and OV-90 cells overexpressing CD157 vs the corresponding mock cells. (DOCX) [file pone.0043649.s006.docx]

Table S3. Genes up-regulated in OVCAR-3 and OV-90 cells overexpressing CD157 *vs* the corresponding mock cells.

|  |  |  | **OVCAR-3** | **OV-90** |
| --- | --- | --- | --- | --- |
| ***Entrez ID*** | ***GeneSymbol*** | ***GeneName*** | ***logFC*** | ***logFC*** |
| **8751** | ADAM15 | ADAM metallopeptidase domain 15 | 0.93 | 1.53 |
| **83543** | AIF1L | allograft inflammatory factor 1-like | 1.41 | 0.80 |
| **50650** | ARHGEF3 | Rho guanine nucleotide exchange factor (GEF) 3 | 1.49 | 0.69 |
| **140462** | ASB9 | ankyrin repeat and SOCS box containing 9 | 2.05 | 1.25 |
| **51761** | ATP8A2 | ATPase, aminophospholipid transporter, class I, type 8A, member 2 | 1.47 | 0.38 |
| **8708** | B3GALT1 | UDP-Gal:betaGlcNAc beta 1,3-galactosyltransferase, polypeptide 1 | 1.30 | 0.38 |
| **655** | BMP7 | bone morphogenetic protein 7 | 4.00 | 1.00 |
| **140707** | BRI3BP | BRI3 binding protein | 1.58 | 0.41 |
| **683** | BST1 | bone marrow stromal cell antigen 1 | 3.42 | 2.50 |
| **118663** | BTBD16 | BTB (POZ) domain containing 16 | 3.35 | 0.55 |
| **55010** | C12orf48 | chromosome 12 open reading frame 48 (PARP-1 binding protein) | 1.05 | 0.57 |
| **80017** | C14orf159 | chromosome 14 open reading frame 159 (UPF0317 protein C14orf159, mitochondrial) | 0.50 | 1.20 |
| **100130933** | C17orf110 | chromosome 17 open reading frame 110 (putative uncharacterized protein C17orf110) | 2.20 | 1.63 |
| **128710** | C20orf94 | chromosome 20 open reading frame 94 (UPF0492 protein C20orf94) | 0.21 | 1.12 |
| **150590** | C2orf15 | chromosome 2 open reading frame 15 (uncharacterized protein C2orf15) | 0.39 | 1.09 |
| **203111** | C8orf47 | chromosome 8 open reading frame 47 (uncharacterized protein C8orf47) | 2.28 | 1.55 |
| **91057** | CCDC34 | coiled-coil domain containing 34 | 0.44 | 1.52 |
| **79937** | CNTNAP3 | contactin associated protein-like 3 | 1.69 | 0.36 |
| **22837** | COBLL1 | COBL-like 1 | 1.20 | 0.76 |
| **1282** | COL4A1 | collagen, type IV, alpha 1 | 1.89 | 0.62 |
| **1284** | COL4A2 | collagen, type IV, alpha 2 | 2.36 | 0.76 |
| **1363** | CPE | carboxypeptidase E | 2.04 | 0.40 |
| **9244** | CRLF1 | cytokine receptor-like factor 1 | 1.40 | 1.83 |
| **54677** | CROT | carnitine O-octanoyltransferase | 0.74 | 1.53 |
| **1466** | CSRP2 | cysteine and glycine-rich protein 2 | 0.51 | 1.42 |
| **56474** | CTPS2 | CTP synthase II | 0.34 | 1.05 |
| **83992** | CTTNBP2 | cortactin binding protein 2 | 4.72 | 0.73 |
| **159013** | CXorf38 | chromosome X open reading frame 38 (uncharacterized protein CXorf38) | 1.25 | 0.38 |
| **51523** | CXXC5 | CXXC finger protein 5 | 1.57 | 2.15 |
| **54541** | DDIT4 | DNA-damage-inducible transcript 4 | 1.98 | 0.61 |
| **1745** | DLX1 | distal-less homeobox 1 | 0.97 | 1.97 |
| **1875** | E2F5 | E2F transcription factor 5, p130-binding | 1.73 | 0.97 |
| **1945** | EFNA4 | ephrin-A4 | 0.90 | 1.28 |
| **1947** | EFNB1 | ephrin-B1 | 1.05 | 1.45 |
| **79767** | ELMO3 | engulfment and cell motility 3 | 1.27 | 0.36 |
| **4072** | EPCAM | epithelial cell adhesion molecule | 5.48 | 0.42 |
| **54869** | EPS8L1 | EPS8-like 1 | 1.20 | 0.48 |
| **79956** | ERMP1 | endoplasmic reticulum metallopeptidase 1 | 1.77 | 0.97 |
| **2114** | ETS2 | v-ets erythroblastosis virus E26 oncogene homolog 2 (avian) | 0.93 | 1.13 |
| **58489** | FAM108C1 | family with sequence similarity 108, member C1 | 0.45 | 1.42 |
| **25854** | FAM149A | family with sequence similarity 149, member A | 1.67 | 0.94 |
| **100131997** | FAM27E3 | family with sequence similarity 27, member E3 | 1.91 | 0.26 |
| **2254** | FGF9 | fibroblast growth factor 9 (glia-activating factor) | 2.23 | 0.97 |
| **2289** | FKBP5 | FK506 binding protein 5 | 0.65 | 1.88 |
| **2348** | FOLR1 | folate receptor 1 (adult) | 2.37 | 0.36 |
| **2308** | FOXO1 | forkhead box O1 | 1.63 | 0.28 |
| **80144** | FRAS1 | Fraser syndrome 1 | 0.73 | 1.06 |
| **2530** | FUT8 | fucosyltransferase 8 (alpha (1,6) fucosyltransferase) | 0.39 | 1.04 |
| **8322** | FZD4 | frizzled family receptor 4 | 2.44 | 0.79 |
| **8324** | FZD7 | frizzled family receptor 7 | 1.59 | 1.76 |
| **2621** | GAS6 | growth arrest-specific 6 | 0.43 | 1.23 |
| **113263** | GLCCI1 | glucocorticoid induced transcript 1 | 0.78 | 1.13 |
| **83468** | GLT8D2 | glycosyltransferase 8 domain containing 2 | 2.40 | 0.92 |
| **2791** | GNG11 | guanine nucleotide binding protein (G protein), gamma 11 | 5.14 | 0.95 |
| **8908** | GYG2 | glycogenin 2 | 1.68 | 1.18 |
| **79366** | HMGN5 | high mobility group nucleosome binding domain 5 | 0.57 | 2.31 |
| **51361** | HOOK1 | hook homolog 1 (Drosophila) | 0.96 | 1.32 |
| **3206** | HOXA10 | homeobox A10 | 2.10 | 0.35 |
| **3213** | HOXB3 | homeobox B3 | 3.54 | 0.34 |
| **3215** | HOXB5 | homeobox B5 | 4.03 | 0.48 |
| **3218** | HOXB8 | homeobox B8 | 2.95 | 0.48 |
| **3219** | HOXB9 | homeobox B9 | 3.22 | 0.92 |
| **9394** | HS6ST1 | heparan sulfate 6-O-sulfotransferase 1 | 1.92 | 0.62 |
| **90161** | HS6ST2 | heparan sulfate 6-O-sulfotransferase 2 | 2.26 | 2.45 |
| **3382** | ICA1 | islet cell autoantigen 1, 69kDa | 0.52 | 2.50 |
| **3418** | IDH2 | isocitrate dehydrogenase 2 (NADP+), mitochondrial | 0.65 | 1.32 |
| **389792** | IER5L | immediate early response 5-like | 1.21 | 2.30 |
| **3485** | IGFBP2 | insulin-like growth factor binding protein 2, 36kDa | 3.36 | 2.65 |
| **140862** | ISM1 | isthmin 1 homolog (zebrafish) | 0.24 | 2.48 |
| **3685** | ITGAV | integrin, alpha V (vitronectin receptor, alpha polypeptide, antigen CD51) | 1.62 | 0.30 |
| **3691** | ITGB4 | integrin, beta 4 | 1.27 | 1.98 |
| **8645** | KCNK5 | potassium channel, subfamily K, member 5 | 1.16 | 1.08 |
| **283102** | KRT8P41 | keratin 8 pseudogene 41 | 2.31 | 0.27 |
| **3898** | LAD1 | ladinin 1 | 3.59 | 3.66 |
| **3902** | LAG3 | lymphocyte-activation gene 3 | 0.56 | 1.74 |
| **3918** | LAMC2 | laminin, gamma 2 | 0.78 | 2.10 |
| **3936** | LCP1 | lymphocyte cytosolic protein 1 (L-plastin) | 0.54 | 1.11 |
| **100505938** | LOC100505938 | hypothetical LOC100505938 | 0.76 | 1.07 |
| **100506305** | LOC100506305 | hypothetical LOC100506305 | 3.21 | 0.49 |
| **645249** | LOC645249 | hypothetical LOC645249 | 0.71 | 1.25 |
| **645722** | LOC645722 | hypothetical protein LOC645722 | 1.68 | 0.40 |
| **23266** | LPHN2 | latrophilin 2 | 2.37 | 1.03 |
| **79694** | MANEA | mannosidase, endo-alpha | 0.50 | 1.23 |
| **149175** | MANEAL | mannosidase, endo-alpha-like | 0.93 | 2.15 |
| **5608** | MAP2K6 | mitogen-activated protein kinase kinase 6 | 1.56 | 0.41 |
| **1953** | MEGF6 | multiple EGF-like-domains 6 | 2.66 | 0.40 |
| **4240** | MFGE8 | milk fat globule-EGF factor 8 protein | 1.50 | 0.79 |
| **3110** | MNX1 | motor neuron and pancreas homeobox 1 | 0.40 | 1.94 |
| **10205** | MPZL2 | myelin protein zero-like 2 | 1.30 | 0.81 |
| **9107** | MTMR6 | myotubularin related protein 6 | 2.56 | 0.27 |
| **4613** | MYCN | v-myc myelocytomatosis viral related oncogene, neuroblastoma derived (avian) | 1.66 | 1.07 |
| **4638** | MYLK | myosin light chain kinase | 1.13 | 0.45 |
| **80896** | NPL | N-acetylneuraminate pyruvate lyase (dihydrodipicolinate synthase) | 1.60 | 0.37 |
| **7025** | NR2F1 | nuclear receptor subfamily 2, group F, member 1 | 3.30 | 1.42 |
| **7026** | NR2F2 | nuclear receptor subfamily 2, group F, member 2 | 1.74 | 0.63 |
| **11163** | NUDT4 | nudix (nucleoside diphosphate linked moiety X)-type motif 4 | 1.78 | 0.45 |
| **9480** | ONECUT2 | one cut homeobox 2 | 1.74 | 0.79 |
| **5140** | PDE3B | phosphodiesterase 3B, cGMP-inhibited | 0.42 | 1.00 |
| **5150** | PDE7A | phosphodiesterase 7A | 0.44 | 1.55 |
| **23037** | PDZD2 | PDZ domain containing 2 | 0.76 | 1.74 |
| **55825** | PECR | peroxisomal trans-2-enoyl-CoA reductase | 1.00 | 0.44 |
| **26207** | PITPNC1 | phosphatidylinositol transfer protein, cytoplasmic 1 | 1.14 | 0.60 |
| **55344** | PLCXD1 | phosphatidylinositol-specific phospholipase C, X domain containing 1 | 3.23 | 0.65 |
| **91584** | PLXNA4 | plexin A4 | 1.95 | 0.40 |
| **5420** | PODXL | podocalyxin-like | 1.89 | 0.33 |
| **29968** | PSAT1 | phosphoserine aminotransferase 1 | 1.36 | 0.74 |
| **5818** | PVRL1 | poliovirus receptor-related 1 (herpesvirus entry mediator C) | 0.77 | 1.44 |
| **26056** | RAB11FIP5 | RAB11 family interacting protein 5 (class I) | 0.35 | 1.14 |
| **5865** | RAB3B | RAB3B, member RAS oncogene family | 0.59 | 1.25 |
| **5891** | RAGE | renal tumor antigen | 1.83 | 0.47 |
| **5983** | RFC3 | replication factor C (activator 1) 3, 38kDa | 0.51 | 1.00 |
| **55819** | RNF130 | ring finger protein 130 | 0.54 | 1.95 |
| **84816** | RTN4IP1 | reticulon 4 interacting protein 1 | 1.12 | 0.29 |
| **6275** | S100A4 | S100 calcium binding protein A4 | 0.29 | 1.75 |
| **6337** | SCNN1A | sodium channel, nonvoltage-gated 1 alpha | 0.59 | 1.79 |
| **57556** | SEMA6A | sema domain, transmembrane domain (TM), and cytoplasmic domain, (semaphorin) 6A | 1.16 | 1.56 |
| **6422** | SFRP1 | secreted frizzled-related protein 1 | 1.20 | 2.16 |
| **157285** | SGK223 | homolog of rat pragma of Rnd2 | 1.28 | 0.48 |
| **124923** | SGK494 | uncharacterized serine/threonine-protein kinase SgK494 | 1.00 | 0.28 |
| **130367** | SGPP2 | sphingosine-1-phosphate phosphatase 2 | 1.79 | 0.98 |
| **6451** | SH3BGRL | SH3 domain binding glutamic acid-rich protein like | 0.92 | 2.66 |
| **387914** | SHISA2 | shisa homolog 2 (Xenopus laevis) | 0.58 | 1.95 |
| **6495** | SIX1 | SIX homeobox 1 | 0.76 | 1.37 |
| **54020** | SLC37A1 | solute carrier family 37 (glycerol-3-phosphate transporter), member 1 | 1.10 | 0.26 |
| **54498** | SMOX | spermine oxidase | 1.01 | 0.33 |
| **10580** | SORBS1 | sorbin and SH3 domain containing 1 | 1.00 | 1.49 |
| **23635** | SSBP2 | single-stranded DNA binding protein 2 | 1.48 | 0.64 |
| **26872** | STEAP1 | six transmembrane epithelial antigen of the prostate 1 | 2.00 | 1.36 |
| **219736** | STOX1 | storkhead box 1 | 2.17 | 0.25 |
| **55359** | STYK1 | serine/threonine/tyrosine kinase 1 | 0.43 | 1.04 |
| **10579** | TACC2 | transforming, acidic coiled-coil containing protein 2 | 0.85 | 1.41 |
| **6948** | TCN2 | transcobalamin II | 1.14 | 0.44 |
| **79600** | TCTN1 | tectonic family member 1 | 1.03 | 0.64 |
| **7980** | TFPI2 | tissue factor pathway inhibitor 2 | 2.05 | 0.77 |
| **84216** | TMEM117 | transmembrane protein 117 | 0.89 | 1.83 |
| **80757** | TMEM121 | transmembrane protein 121 | 1.39 | 0.24 |
| **92691** | TMEM169 | transmembrane protein 169 | 1.31 | 0.38 |
| **201931** | TMEM192 | transmembrane protein 192 | 0.56 | 1.58 |
| **7114** | TMSB4X | thymosin beta 4, X-linked | 0.82 | 1.23 |
| **8718** | TNFRSF25 | tumor necrosis factor receptor superfamily, member 25 | 1.74 | 0.28 |
| **10040** | TOM1L1 | target of myb1 (chicken)-like 1 | 0.37 | 1.22 |
| **11257** | TP53TG1 | TP53 target 1 (non-protein coding) | 0.35 | 2.15 |
| **7164** | TPD52L1 | tumor protein D52-like 1 | 1.94 | 3.99 |
| **7168** | TPM1 | tropomyosin 1 (alpha) | 1.79 | 1.18 |
| **6738** | TROVE2 | TROVE domain family, member 2 | 0.58 | 1.00 |
| **10194** | TSHZ1 | teashirt zinc finger homeobox 1 | 1.71 | 0.98 |
| **7365** | UGT2B10 | UDP glucuronosyltransferase 2 family, polypeptide B10 | 1.45 | 0.25 |
| **10720** | UGT2B11 | UDP glucuronosyltransferase 2 family, polypeptide B11 | 1.36 | 0.42 |
| **7364** | UGT2B7 | UDP glucuronosyltransferase 2 family, polypeptide B7 | 1.54 | 0.38 |
| **10451** | VAV3 | vav 3 guanine nucleotide exchange factor | 1.70 | 0.30 |
| **1462** | VCAN | versican | 3.57 | 0.37 |
| **9686** | VGLL4 | vestigial like 4 (Drosophila) | 0.87 | 1.21 |
| **79971** | WLS | wntless homolog (Drosophila) | 2.07 | 0.76 |
| **80326** | WNT10A | wingless-type MMTV integration site family, member 10A | 0.55 | 1.21 |
| **7475** | WNT6 | wingless-type MMTV integration site family, member 6 | 1.71 | 1.45 |
| **284273** | ZADH2 | zinc binding alcohol dehydrogenase domain containing 2 | 1.48 | 1.44 |
| **29800** | ZDHHC1 | zinc finger, DHHC-type containing 1 | 0.41 | 1.55 |
| **150244** | ZDHHC8P1 | zinc finger, DHHC-type containing 8 pseudogene 1 | 2.04 | 1.11 |
| **85416** | ZIC5 | Zic family member 5 | 1.22 | 0.30 |
| **7581** | ZNF33A | zinc finger protein 33A | 0.27 | 1.45 |
| **84671** | ZNF347 | zinc finger protein 347 | 0.56 | 1.17 |
| **59348** | ZNF350 | zinc finger protein 350 | 1.74 | 0.44 |
| **79986** | ZNF702P | zinc finger protein 702, pseudogene | 1.22 | 1.60 |
