# Supplementary material for: Overexpression of CD157 Contributes to Epithelial Ovarian Cancer Progression by Promoting Mesenchymal Differentiation
Source: PLoS One. 2012 Aug 20;7(8):e43649. doi: 10.1371/journal.pone.0043649 (PMC3423388; doi:10.1371/journal.pone.0043649)
Supplement: Table S4 — Genes down-regulated in OVCAR-3 and OV-90 cells overexpressing CD157 vs the corresponding control cells. (DOCX) [file pone.0043649.s007.docx]

Table S4. Genes down-regulated in OVCAR-3 and OV-90 cells overexpressing CD157 *vs* the corresponding control cells.

|  |  |  | **OVCAR-3** | **OV-90** |
| --- | --- | --- | --- | --- |
| ***Entrez ID*** | ***GeneSymbol*** | ***GeneName*** | ***logFC*** | ***logFC*** |
| **9510** | ADAMTS1 | ADAM metallopeptidase with thrombospondin type 1 motif, 1 | -0.71 | -1.81 |
| **133** | ADM | adrenomedullin | -2.89 | -1.51 |
| **11217** | AKAP2 | A kinase (PRKA) anchor protein 2 | -2.66 | -0.63 |
| **4329** | ALDH6A1 | aldehyde dehydrogenase 6 family, member A1 | -0.67 | -1.04 |
| **241** | ALOX5AP | arachidonate 5-lipoxygenase-activating protein | -0.55 | -1.64 |
| **651746** | ANKRD33B | ankyrin repeat domain 33B | -1.02 | -0.57 |
| **65124** | ANKRD57 | ankyrin repeat domain 57 | -1.53 | -0.65 |
| **83464** | APH1B | anterior pharynx defective 1 homolog B (C. elegans) | -0.64 | -2.59 |
| **8542** | APOL1 | apolipoprotein L, 1 | -2.20 | -0.77 |
| **10865** | ARID5A | AT rich interactive domain 5A (MRF1-like) | -1.04 | -0.35 |
| **80117** | ARL14 | ADP-ribosylation factor-like 14 | -1.42 | -0.27 |
| **558** | AXL | AXL receptor tyrosine kinase | -1.36 | -0.36 |
| **570** | BAAT | bile acid CoA: amino acid N-acyltransferase (glycine N-choloyltransferase) | -0.53 | -1.10 |
| **597** | BCL2A1 | BCL2-related protein A1 | -0.51 | -1.06 |
| **650** | BMP2 | bone morphogenetic protein 2 | -2.50 | -1.23 |
| **664** | BNIP3 | BCL2/adenovirus E1B 19kDa interacting protein 3 | -0.73 | -2.32 |
| **56935** | C11orf75 | chromosome 11 open reading frame 75 (UPF0443 protein C11orf75) | -1.05 | -0.74 |
| **55337** | C19orf66 | chromosome 19 open reading frame 66 (UPF0515 protein C19orf66) | -1.24 | -0.60 |
| **716** | C1S | complement component 1, s subcomponent | -0.39 | -1.13 |
| **152641** | C4orf38 | chromosome 4 open reading frame 38 | -1.87 | -0.44 |
| **221749** | C6orf145 | chromosome 6 open reading frame 145 | -1.29 | -0.72 |
| **286144** | C8orf83 | chromosome 8 open reading frame 83 | -0.57 | -1.62 |
| **84267** | C9orf64 | chromosome 9 open reading frame 64 | -0.38 | -1.30 |
| **114769** | CARD16 | caspase recruitment domain family, member 16 | -0.94 | -1.08 |
| **834** | CASP1 | caspase 1, apoptosis-related cysteine peptidase (interleukin 1, beta, convertase) | -1.23 | -0.50 |
| **837** | CASP4 | caspase 4, apoptosis-related cysteine peptidase | -3.75 | -0.85 |
| **838** | CASP5 | caspase 5, apoptosis-related cysteine peptidase | -3.15 | -0.77 |
| **842** | CASP9 | caspase 9, apoptosis-related cysteine peptidase | -0.26 | -1.03 |
| **25819** | CCRN4L | CCR4 carbon catabolite repression 4-like (S. cerevisiae) | -1.05 | -0.39 |
| **23607** | CD2AP | CD2-associated protein | -0.71 | -1.01 |
| **1604** | CD55 | CD55 molecule, decay accelerating factor for complement (Cromer blood group) | -2.15 | -0.61 |
| **968** | CD68 | CD68 molecule | -1.20 | -0.33 |
| **10602** | CDC42EP3 | CDC42 effector protein (Rho GTPase binding) 3 | -1.89 | -0.81 |
| **55602** | CDKN2AIP | CDKN2A interacting protein | -1.04 | -0.99 |
| **84952** | CGNL1 | cingulin-like 1 | -1.10 | -1.78 |
| **26511** | CHIC2 | cysteine-rich hydrophobic domain 2 | -0.79 | -1.06 |
| **1119** | CHKA | choline kinase alpha | -1.73 | -0.39 |
| **51200** | CPA4 | carboxypeptidase A4 | -1.68 | -2.07 |
| **126129** | CPT1C | carnitine palmitoyltransferase 1C | -0.71 | -1.83 |
| **54504** | CPVL | carboxypeptidase, vitellogenic-like | -1.82 | -0.31 |
| **84699** | CREB3L3 | cAMP responsive element binding protein 3-like 3 | -0.35 | -1.44 |
| **51232** | CRIM1 | cysteine rich transmembrane BMP regulator 1 (chordin-like) | -1.85 | -0.79 |
| **1396** | CRIP1 | cysteine-rich protein 1 (intestinal) | -0.36 | -1.03 |
| **1519** | CTSO | cathepsin O | -0.47 | -1.58 |
| **2920** | CXCL2 | chemokine (C-X-C motif) ligand 2 | -2.21 | -0.41 |
| **2921** | CXCL3 | chemokine (C-X-C motif) ligand 3 | -2.25 | -0.45 |
| **56603** | CYP26B1 | cytochrome P450, family 26, subfamily B, polypeptide 1 | -1.00 | -1.31 |
| **115265** | DDIT4L | DNA-damage-inducible transcript 4-like | -1.29 | -1.94 |
| **91351** | DDX60L | DEAD (Asp-Glu-Ala-Asp) box polypeptide 60-like | -0.37 | -1.27 |
| **10202** | DHRS2 | dehydrogenase/reductase (SDR family) member 2 | -0.98 | -1.62 |
| **10901** | DHRS4 | dehydrogenase/reductase (SDR family) member 4 | -0.78 | -1.52 |
| **728635** | DHRS4L1 | dehydrogenase/reductase (SDR family) member 4 like 1 | -0.51 | -1.09 |
| **1789** | DNMT3B | DNA (cytosine-5-)-methyltransferase 3 beta | -2.38 | -0.25 |
| **1843** | DUSP1 | dual specificity phosphatase 1 | -2.47 | -0.26 |
| **11221** | DUSP10 | dual specificity phosphatase 10 | -1.63 | -0.26 |
| **51207** | DUSP13 | dual specificity phosphatase 13 | -0.32 | -2.53 |
| **11072** | DUSP14 | dual specificity phosphatase 14 | -1.48 | -0.81 |
| **1906** | EDN1 | endothelin 1 | -0.39 | -1.70 |
| **30846** | EHD2 | EH-domain containing 2 | -2.01 | -1.96 |
| **2013** | EMP2 | epithelial membrane protein 2 | -2.49 | -0.91 |
| **2066** | ERBB4 | v-erb-a erythroblastic leukemia viral oncogene homolog 4 (avian) | -0.40 | -2.67 |
| **3992** | FADS1 | fatty acid desaturase 1 | -0.48 | -1.29 |
| **9415** | FADS2 | fatty acid desaturase 2 | -1.21 | -0.95 |
| **159091** | FAM122C | family with sequence similarity 122C | -0.29 | -2.19 |
| **116496** | FAM129A | family with sequence similarity 129, member A | -4.03 | -1.33 |
| **54463** | FAM134B | family with sequence similarity 134, member B | -1.80 | -2.98 |
| **55603** | FAM46A | family with sequence similarity 46, member A | -1.49 | -1.13 |
| **115572** | FAM46B | family with sequence similarity 46, member B | -1.01 | -0.62 |
| **81553** | FAM49A | family with sequence similarity 49, member A | -1.97 | -0.40 |
| **2200** | FBN1 | fibrillin 1 | -2.40 | -1.45 |
| **26269** | FBXO8 | F-box protein 8 | -1.28 | -0.79 |
| **57600** | FNIP2 | folliculin interacting protein 2 | -1.54 | -1.53 |
| **2353** | FOS | FBJ murine osteosarcoma viral oncogene homolog | -1.36 | -0.99 |
| **51083** | GAL | galanin prepropeptide | -1.23 | -0.42 |
| **2627** | GATA6 | GATA binding protein 6 | -1.42 | -1.74 |
| **2643** | GCH1 | GTP cyclohydrolase 1 | -0.31 | -1.26 |
| **2710** | GK | glycerol kinase | -1.39 | -0.33 |
| **2982** | GUCY1A3 | guanylate cyclase 1, soluble, alpha 3 | -0.54 | -1.18 |
| **2983** | GUCY1B3 | guanylate cyclase 1, soluble, beta 3 | -1.50 | -1.91 |
| **51454** | GULP1 | GULP, engulfment adaptor PTB domain containing 1 | -2.64 | -1.14 |
| **3005** | H1F0 | H1 histone family, member 0 | -1.06 | -0.46 |
| **57493** | HEG1 | HEG homolog 1 (zebrafish) | -1.24 | -0.35 |
| **9931** | HELZ | helicase with zinc finger | -1.09 | -0.33 |
| **8334** | HIST1H2AC | histone cluster 1, H2ac | -1.81 | -0.62 |
| **3013** | HIST1H2AD | histone cluster 1, H2ad | -2.08 | -0.34 |
| **3012** | HIST1H2AE | histone cluster 1, H2ae | -1.59 | -0.34 |
| **8344** | HIST1H2BE | histone cluster 1, H2be | -2.02 | -0.26 |
| **8345** | HIST1H2BH | histone cluster 1, H2bh | -2.11 | -0.24 |
| **8346** | HIST1H2BI | histone cluster 1, H2bi | -1.91 | -0.26 |
| **8970** | HIST1H2BJ | histone cluster 1, H2bj | -2.09 | -0.25 |
| **85236** | HIST1H2BK | histone cluster 1, H2bk | -2.57 | -0.38 |
| **8340** | HIST1H2BL | histone cluster 1, H2bl | -2.21 | -0.25 |
| **8351** | HIST1H3D | histone cluster 1, H3d | -1.48 | -0.34 |
| **8365** | HIST1H4H | histone cluster 1, H4h | -1.65 | -0.25 |
| **723790** | HIST2H2AA4 | histone cluster 2, H2aa4 | -2.32 | -0.37 |
| **8349** | HIST2H2BE | histone cluster 2, H2be | -2.10 | -0.63 |
| **3294** | HSD17B2 | hydroxysteroid (17-beta) dehydrogenase 2 | -3.09 | -1.40 |
| **3373** | HYAL1 | hyaluronoglucosaminidase 1 | -1.76 | -0.29 |
| **3384** | ICAM2 | intercellular adhesion molecule 2 | -1.05 | -0.67 |
| **3569** | IL6 | interleukin 6 (interferon, beta 2) | -0.66 | -3.01 |
| **9314** | KLF4 | Kruppel-like factor 4 (gut) | -0.48 | -1.44 |
| **1316** | KLF6 | Kruppel-like factor 6 | -0.60 | -1.50 |
| **388533** | KRTDAP | keratinocyte differentiation-associated protein | -0.20 | -1.70 |
| **81606** | LBH | limb bud and heart development homolog (mouse) | -2.27 | -1.35 |
| **10186** | LHFP | lipoma HMGIC fusion partner | -0.92 | -1.99 |
| **3977** | LIFR | leukemia inhibitory factor receptor alpha | -1.61 | -0.89 |
| **100128893** | LOC100128893 | hypothetical LOC100128893 | -0.29 | -1.58 |
| **100288911** | LOC100288911 | hypothetical LOC100288911 | -2.21 | -1.07 |
| **100506870** | LOC100506870 | hypothetical LOC100506870 | -1.96 | -0.50 |
| **100506990** | LOC100506990 | hypothetical LOC100506990 | -0.38 | -2.26 |
| **100510161** | LOC100510161 | hypothetical LOC100510161 | -1.33 | -0.24 |
| **151534** | LOC151534 | hypothetical LOC151534 | -1.10 | -0.43 |
| **643650** | LOC643650 | hypothetical LOC643650 | -1.40 | -0.54 |
| **23175** | LPIN1 | lipin 1 | -1.51 | -1.51 |
| **84230** | LRRC8C | leucine rich repeat containing 8 family, member C | -1.97 | -0.74 |
| **129530** | LYG1 | lysozyme G-like 1 | -1.10 | -0.48 |
| **23764** | MAFF | v-maf musculoaponeurotic fibrosarcoma oncogene homolog F (avian) | -1.01 | -0.45 |
| **81631** | MAP1LC3B | microtubule-associated protein 1 light chain 3 beta | -0.66 | -1.26 |
| **79884** | MAP9 | microtubule-associated protein 9 | -1.36 | -2.44 |
| **4082** | MARCKS | myristoylated alanine-rich protein kinase C substrate | -3.38 | -0.68 |
| **4147** | MATN2 | matrilin 2 | -0.91 | -1.43 |
| **10150** | MBNL2 | muscleblind-like 2 (Drosophila) | -1.09 | -0.87 |
| **1955** | MEGF9 | multiple EGF-like-domains 9 | -2.11 | -0.44 |
| **4257** | MGST1 | microsomal glutathione S-transferase 1 | -4.37 | -0.26 |
| **9645** | MICAL2 | microtubule associated monoxygenase, calponin and LIM domain containing 2 | -1.40 | -0.55 |
| **4286** | MITF | microphthalmia-associated transcription factor | -0.82 | -1.62 |
| **54996** | MOSC2 | MOCO sulphurase C-terminal domain containing 2 | -0.55 | -2.02 |
| **56180** | MOSPD1 | motile sperm domain containing 1 | -0.57 | -1.62 |
| **8777** | MPDZ | multiple PDZ domain protein | -1.71 | -1.76 |
| **4488** | MSX2 | msh homeobox 2 | -1.60 | -0.91 |
| **55545** | MSX2P1 | msh homeobox 2 pseudogene 1 | -1.48 | -0.53 |
| **10588** | MTHFS | 5,10-methenyltetrahydrofolate synthetase (5-formyltetrahydrofolate cyclo-ligase) | -0.53 | -1.87 |
| **4615** | MYD88 | myeloid differentiation primary response gene (88) | -1.55 | -0.36 |
| **10398** | MYL9 | myosin, light chain 9, regulatory | -0.76 | -1.43 |
| **135112** | NCOA7 | nuclear receptor coactivator 7 | -0.87 | -1.96 |
| **90271** | NCRNA00263 | non-protein coding RNA 263 | -0.41 | -1.57 |
| **4535** | ND1 | NADH dehydrogenase, subunit 1 (complex I) | -1.32 | -0.33 |
| **4541** | ND6 | NADH dehydrogenase, subunit 6 (complex I) | -1.63 | -1.77 |
| **4824** | NKX3-1 | NK3 homeobox 1 | -2.30 | -1.15 |
| **57502** | NLGN4X | neuroligin 4, X-linked | -1.66 | -0.49 |
| **4879** | NPPB | natriuretic peptide B | -1.90 | -0.32 |
| **3084** | NRG1 | neuregulin 1 | -1.82 | -0.87 |
| **146183** | OTOA | otoancorin | -0.24 | -1.32 |
| **9060** | PAPSS2 | 3'-phosphoadenosine 5'-phosphosulfate synthase 2 | -0.46 | -1.15 |
| **56965** | PARP6 | poly (ADP-ribose) polymerase family, member 6 | -0.34 | -1.86 |
| **5090** | PBX3 | pre-B-cell leukemia homeobox 3 | -1.75 | -0.71 |
| **56034** | PDGFC | platelet derived growth factor C | -2.93 | -1.12 |
| **64236** | PDLIM2 | PDZ and LIM domain 2 (mystique) | -1.27 | -0.49 |
| **118987** | PDZD8 | PDZ domain containing 8 | -1.05 | -0.28 |
| **5236** | PGM1 | phosphoglucomutase 1 | -0.83 | -1.28 |
| **5266** | PI3 | peptidase inhibitor 3, skin-derived | -1.35 | -1.19 |
| **5292** | PIM1 | pim-1 oncogene | -0.31 | -1.65 |
| **5327** | PLAT | plasminogen activator, tissue | -1.11 | -1.50 |
| **79887** | PLBD1 | phospholipase B domain containing 1 | -0.99 | -2.18 |
| **5352** | PLOD2 | procollagen-lysine, 2-oxoglutarate 5-dioxygenase 2 | -1.35 | -0.53 |
| **5360** | PLTP | phospholipid transfer protein | -0.24 | -1.15 |
| **5460** | POU5F1 | POU class 5 homeobox 1 | -2.07 | -0.47 |
| **642559** | POU5F1P3 | POU class 5 homeobox 1 pseudogene 3 | -2.05 | -0.35 |
| **645682** | POU5F1P4 | POU class 5 homeobox 1 pseudogene 4 | -1.96 | -0.39 |
| **25845** | PP7080 | hypothetical LOC25845 | -0.51 | -1.76 |
| **8496** | PPFIBP1 | PTPRF interacting protein, binding protein 1 (liprin beta 1) | -0.73 | -1.75 |
| **23645** | PPP1R15A | protein phosphatase 1, regulatory (inhibitor) subunit 15A | -2.08 | -0.47 |
| **5538** | PPT1 | palmitoyl-protein thioesterase 1 | -1.26 | -0.53 |
| **8842** | PROM1 | prominin 1 | -1.55 | -3.23 |
| **5734** | PTGER4 | prostaglandin E receptor 4 (subtype EP4) | -1.03 | -1.36 |
| **754** | PTTG1IP | pituitary tumor-transforming 1 interacting protein | -1.13 | -0.54 |
| **11264** | PXMP4 | peroxisomal membrane protein 4, 24kDa | -1.46 | -0.37 |
| **23475** | QPRT | quinolinate phosphoribosyltransferase | -1.04 | -0.84 |
| **11031** | RAB31 | RAB31, member RAS oncogene family | -0.27 | -1.20 |
| **10981** | RAB32 | RAB32, member RAS oncogene family | -3.75 | -0.92 |
| **10267** | RAMP1 | receptor (G protein-coupled) activity modifying protein 1 | -0.47 | -2.43 |
| **9693** | RAPGEF2 | Rap guanine nucleotide exchange factor (GEF) 2 | -0.62 | -1.31 |
| **65059** | RAPH1 | Ras association (RalGDS/AF-6) and pleckstrin homology domains 1 | -2.17 | -0.45 |
| **23180** | RFTN1 | raftlin, lipid raft linker 1 | -2.39 | -1.07 |
| **26575** | RGS17 | regulator of G-protein signaling 17 | -1.04 | -1.59 |
| **5997** | RGS2 | regulator of G-protein signaling 2, 24kDa | -1.43 | -1.74 |
| **8490** | RGS5 | regulator of G-protein signaling 5 | -0.28 | -3.69 |
| **9886** | RHOBTB1 | Rho-related BTB domain containing 1 | -1.62 | -0.74 |
| **29984** | RHOD | ras homolog gene family, member D | -0.56 | -1.20 |
| **83547** | RILP | Rab interacting lysosomal protein | -1.16 | -0.62 |
| **8780** | RIOK3 | RIO kinase 3 (yeast) | -0.94 | -1.17 |
| **390** | RND3 | Rho family GTPase 3 | -1.38 | -0.44 |
| **1992** | SERPINB1 | serpin peptidase inhibitor, clade B (ovalbumin), member 1 | -0.45 | -1.12 |
| **5269** | SERPINB6 | serpin peptidase inhibitor, clade B (ovalbumin), member 6 | -0.92 | -1.34 |
| **57568** | SIPA1L2 | signal-induced proliferation-associated 1 like 2 | -2.04 | -0.39 |
| **23411** | SIRT1 | sirtuin 1 | -1.08 | -0.30 |
| **8273** | SLC10A3 | solute carrier family 10 (sodium/bile acid cotransporter family), member 3 | -0.25 | -1.25 |
| **201780** | SLC10A4 | solute carrier family 10 (sodium/bile acid cotransporter family), member 4 | -2.14 | -1.49 |
| **114789** | SLC25A25 | solute carrier family 25 (mitochondrial carrier; phosphate carrier), member 25 | -1.24 | -0.32 |
| **8651** | SOCS1 | suppressor of cytokine signaling 1 | -0.50 | -1.30 |
| **6775** | STAT4 | signal transducer and activator of transcription 4 | -0.32 | -1.04 |
| **23345** | SYNE1 | spectrin repeat containing, nuclear envelope 1 | -1.74 | -1.72 |
| **23224** | SYNE2 | spectrin repeat containing, nuclear envelope 2 | -1.20 | -0.25 |
| **7078** | TIMP3 | TIMP metallopeptidase inhibitor 3 | -1.91 | -1.09 |
| **7088** | TLE1 | transducin-like enhancer of split 1 (E(sp1) homolog, Drosophila) | -1.96 | -0.40 |
| **83935** | TMEM133 | transmembrane protein 133 | -1.03 | -0.53 |
| **134285** | TMEM171 | transmembrane protein 171 | -1.04 | -0.63 |
| **23670** | TMEM2 | transmembrane protein 2 | -1.30 | -0.41 |
| **79041** | TMEM38A | transmembrane protein 38A | -0.61 | -1.31 |
| **120224** | TMEM45B | transmembrane protein 45B | -1.00 | -1.38 |
| **8744** | TNFSF9 | tumor necrosis factor (ligand) superfamily, member 9 | -1.46 | -0.31 |
| **9540** | TP53I3 | tumor protein p53 inducible protein 3 | -1.89 | -1.14 |
| **8460** | TPST1 | tyrosylprotein sulfotransferase 1 | -0.52 | -1.16 |
| **7103** | TSPAN8 | tetraspanin 8 | -0.36 | -1.59 |
| **10382** | TUBB4 | tubulin, beta 4 | -0.90 | -1.13 |
| **84617** | TUBB6 | tubulin, beta 6 | -1.16 | -1.71 |
| **7286** | TUFT1 | tuftelin 1 | -1.79 | -0.24 |
| **26609** | VCX | variable charge, X-linked | -0.47 | -3.87 |
| **51480** | VCX2 | variable charge, X-linked 2 | -0.46 | -3.87 |
| **51481** | VCX3A | variable charge, X-linked 3A | -0.60 | -3.93 |
| **80014** | WWC2 | WW and C2 domain containing 2 | -1.13 | -1.15 |
| **55625** | ZDHHC7 | zinc finger, DHHC-type containing 7 | -0.65 | -1.34 |
| **7570** | ZNF22 | zinc finger protein 22 (KOX 15) | -1.07 | -0.82 |
| **147947** | ZNF542 | zinc finger protein 542 | -0.38 | -1.53 |
| **7784** | ZP3 | zona pellucida glycoprotein 3 (sperm receptor) | -1.31 | -0.49 |
