# Supplementary material for: Overexpression of CD157 Contributes to Epithelial Ovarian Cancer Progression by Promoting Mesenchymal Differentiation
Source: PLoS One. 2012 Aug 20;7(8):e43649. doi: 10.1371/journal.pone.0043649 (PMC3423388; doi:10.1371/journal.pone.0043649)
Supplement: Table S5 — GeneGo pathway maps and GeneGo process networks analysis of the CD157-associated molecular signature. (DOCX) [file pone.0043649.s008.docx]

**Table S5. GeneGo pathway maps and GeneGo process networks analysis of the CD157-associated molecular signature.**

| ***GeneGo pathway maps*** | ***P value*** | ***Ratio*** | ***Genes*** |
| --- | --- | --- | --- |
| GTP metabolism | 6.76E-03 | 0.125 | GUCY1A3, GUCY1B3 |
| Cytoskeleton remodeling_TGF, WNT and cytoskeletal remodeling | 1.55E-02 | 0.075 | CASP9, COL4A1, COL4A2, FZD4, FZD7, MYL9, MYLK, PLAT, WNT10A, WNT6 |
| Cell adhesion_ECM remodeling | 1.85E-02 | 0.098 | COL4A1, COL4A2, ERBB4, LAMC2, PLAT, TIMP3, VCAN |
| Cell adhesion_Plasmin signaling | 1.87E-02 | 0.118 | COL4A1, COL4A2, MAP2K6, PLAT, TFPI2 |
| Nicotine metabolism in liver | 2.88E-02 | 0.222 | UGT2B10, UGT2B7 |
| Immune response_IL-1 signaling pathway | 4.35E-02 | 0.091 | FOS, EDN1, IL6, MAP2K6, MYD88 |
| Cell adhesion_Integrin-mediated cell adhesion and migration | 4.67E-02 | 0.089 | COL4A1, COL4A2, MYLK, MYL9 |
| Cytoskeleton remodeling_Alpha-1A adrenergic receptor-dependent inhibition of PI3K | 4.97E-02 | 0.167 | MYLK, MYL9 |

| ***GeneGO process networks*** | ***P value*** | ***Ratio*** | ***Genes*** |
| --- | --- | --- | --- |
| Development_Ossification and bone remodeling | 5.07E-04 | 0.079 | BMP2, BMP7, ETS2, FOXO1, FZD4, FZD7, IGFBP2, ITGAV, MAP2K6, MSX2, PTGER4, WNT10A, WNT6 |
| Signal transduction_NOTCH signaling | 5.71E-03 | 0.057 | APH1B, ERBB4, FOS, FZD4, FZD7, MAP2K6, NRG1, PDGFC, SFRP1, TLE1, WNT10A, WNT6 |
| Cell adhesion_Cell-matrix interactions | 7.41E-03 | 0.057 | ADAM15, ADAMTS1, COL4A1, COL4A2, FBN1, ITGAV, ITGB4, LAMC2, TIMP3, VCAN |
| Proteolysis_Connective tissue degradation | 1.13E-02 | 0.067 | ADAM15, ADAMTS1, COL4A1, COL4A2, CTSO, LAMC2, PLAT, SERPINB6, TFPI2, TIMP3 |
| Development_Skeletal muscle development | 1.17E-02 | 0.063 | COL4A1, COL4A2, GATA6, MYL9, SIRT1, TPM1 |
| Apoptosis_Death Domain receptors & caspases in apoptosis | 1.37E-02 | 0.065 | CARD16, CASP1, CASP4, CASP5, CASP9, TIMP3, TNFRSF25, TNFSF9 |
| Cell adhesion_Integrin-mediated cell-matrix adhesion | 1.92E-02 | 0.052 | COL4A1, COL4A2, FBN1, ITGAV, ITGB4, LAMC2, MYL9, MYLK, RAPH1, RND3, TSPAN8, TUBB4, TUBB6 |
| Signal Transduction_BMP and GDF signaling | 2.93E-02 | 0.066 | BMP2, BMP7, MAP2K6, MSX2, POU5F1, TLE1 |
| Cell adhesion_Platelet-endothelium-leucocyte interactions | 3.29E-02 | 0.052 | CD68, COL4A1, COL4A2, EDN1, EFNA4, EFNB1, IL6, ITGAV, PDGFC, PLAT, TFPI2 |
| Development_EMT_Regulation of epithelial-to-mesenchymal transition | 4.27E-02 | 0.048 | APH1B, BMP2, BMP7, EDN1, FOS, FZD4, FZD7, HSD17B2, ITGAV, LIFR, MAP2K6, S100A4, TPM1, WNT10A, WNT6 |
| Development_Blood vessel morphogenesis | 4.63E-02 | 0.047 | APH1B, EDN1, ERBB4, FOS, FOXO1, NPPB, NRG1, PDE3B, PDE7A, PLAT |
| Apoptosis_Apoptosis stimulation by external signals | 4.82E-02 | 0.054 | APH1B, CASP9, ERBB4, FOS, NRG1, TNFRSF25 |
